# Expression of the ZNT1 Zinc Transporter from the Metal Hyperaccumulator *Noccaea caerulescens* Confers Enhanced Zinc and Cadmium Tolerance and Accumulation to *Arabidopsis thaliana*

**DOI:** 10.1371/journal.pone.0149750

**Published:** 2016-03-01

**Authors:** Ya-Fen Lin, Zeshan Hassan, Sangita Talukdar, Henk Schat, Mark G. M. Aarts

**Affiliations:** 1 Laboratory of Genetics, Wageningen University and Research Centre, Droevendaalsesteeg 1, 6708 PB, Wageningen, The Netherlands; 2 Institute of Molecular and Cellular Biology, Free University of Amsterdam, De Boelelaan 1085, NL-1081 HV, Amsterdam, The Netherlands; United States Department of Agriculture, Agricultural Research Service, UNITED STATES

## Abstract

Prompt regulation of transition metal transporters is crucial for plant zinc homeostasis. NcZNT1 is one of such transporters, found in the metal hyperaccumulator Brassicaceae species *Noccaea caerulescens*. It is orthologous to AtZIP4 from *Arabidopsis thaliana*, an important actor in Zn homeostasis. We examined if the NcZNT1 function contributes to the metal hyperaccumulation of *N*. *caerulescens*. NcZNT1 was found to be a plasma-membrane located metal transporter. Constitutive overexpression of NcZNT1 in *A*. *thaliana* conferred enhanced tolerance to exposure to excess Zn and Cd supply, as well as increased accumulation of Zn and Cd and induction of the Fe deficiency response, when compared to non-transformed wild-type plants. Promoters of both genes were induced by Zn deficiency in roots and shoots of *A*. *thaliana*. In *A*. *thaliana*, the *AtZIP4* and *NcZNT1* promoters were mainly active in cortex, endodermis and pericycle cells under Zn deficient conditions. In *N*. *caerulescens*, the promoters were active in the same tissues, though the activity of the *NcZNT1* promoter was higher and not limited to Zn deficient conditions. Common *cis* elements were identified in both promoters by 5’ deletion analysis. These correspond to the previously determined Zinc Deficiency Responsive Elements found in *A*. *thaliana* to interact with two redundantly acting transcription factors, bZIP19 and bZIP23, controlling the Zn deficiency response. In conclusion, these results suggest that *NcZNT1* is an important factor in contributing to Zn and Cd hyperaccumulation in *N*. *caerulescens*. Differences in *cis*- and *trans*-regulators are likely to account for the differences in expression between *A*. *thaliana* and *N*. *caerulescens*. The high, constitutive NcZNT1 expression in the stele of *N*. *caerulescens* roots implicates its involvement in long distance root-to-shoot metal transport by maintaining a Zn/Cd influx into cells responsible for xylem loading.

## Introduction

Zinc (Zn) is an essential component of several important enzymes in plants, such as RNA polymerase, alcohol dehydrogenase, Cu/Zn superoxide dismutase and carbonic anhydrase [1, 2]. In addition, many proteins contain Zn in structural domains, eg. in Zn finger domains. Poor growth and reduced biomass are among the major Zn deficiency symptoms that lead to reduced crop yields [1]. Although Zn is essential for plants, elevated concentrations of Zn are generally toxic, causing leaf chlorosis and growth reduction. The toxic effects are due to uncontrolled binding of Zn to proteins and cofactors that renders them non-functional [3]. Excess Zn also leads to a high build-up of damaging Reactive Oxygen Species (ROS) in plant cells [4]. Cadmium (Cd) is a non-essential element with no known biological function in plants. It can be taken up by transporters of minerals which are chemically similar to Cd, such as Fe or Zn [5]. Cd interferes with the DNA repair mechanism and the photosynthesis machinery. Exposure leads to reduced water and nutrient uptake, lowered photosynthesis and ultimately leaf chlorosis and a general reduction in plant growth [6, 7]. It also induces the production of ROS [8]. In response to fluctuations in metal supply concentrations, metal homeostasis mechanisms evolved in plants, which ensure proper regulation of their cellular and organellar metal concentrations to maintain a stable and constant condition [9].

Zn hyperaccumulator species accumulate more than 10,000 μg Zn g^-1^ of dry weight (dw) (1%, w/w) in their leaves [10]. Most plants contain between 30 and 100 μg Zn g^-1^ dw, and concentrations above 300 μg Zn g^-1^ dw are generally toxic [1]. Plants accumulating more than 100 μg Cd g^-1^ dw are considered to be Cd hyperaccumulators [10]. Another characteristic of Zn/Cd hyperaccumulators is that the highest Zn or Cd concentrations are found in the shoots rather than in the roots, whereas generally plants store excess metals in roots, presumably to avoid negative interference with photosynthesis. Two Zn/Cd hyperaccumulators, *Noccaea caerulescens* (previously known as *Thlaspi caerulescens*) and *Arabidopsis halleri*, both belong to the Brassicaceae family. Transcriptional analysis revealed differences in the regulation of genes normally induced by Zn deficiency in these plants. These are often expressed at higher levels and much less transcriptionally responsive to Zn deficiency than in related non-hyperaccumulators [11–13].

Understanding the mode of action of plant Zn/Cd hyperaccumulation is interesting for reasons of evolution and applied biology. Zn/Cd hyperaccumulation evolved independently in the *Arabidopsis* and *Noccaea* genera from a non-hyperaccumulating ancestor [14]. Understanding such convergent evolution at the molecular level will provide insights in the mechanisms that underwent adaptive evolutionary change in both genera. The applied interest in Zn/Cd hyperaccumulators lies in their use for the remediation of metal-polluted soils in a process known as phytoremediation [15]. A disadvantage of the plant species that are currently considered for use in Zn/Cd phytoremediation is that either their biomass is insufficient, or their metal extraction capacity is too low to support economically viable phytoremediation projects [9]. Another application lies in the biofortification of crops. This involves the fascinating concept of correcting human micronutrient deficiencies (including those of Zn and Fe) by developing crops with enhanced contents of bioavailable micronutrients [16]. With increased knowledge on the molecular mechanisms involving Zn and Cd uptake, translocation and accumulation in hyperaccumulating species, it may be possible to engineer Zn/Cd hyperaccumulation and -tolerance in a high-biomass species for Zn/Cd phytoremediation, or to select for crop varieties useful for Zn biofortification purposes, i.e. with low affinity for Cd uptake.

Previously we cloned the *ZNT1* gene from *N*. *caerulescens* (*NcZNT1*), encoding a ZIP-like transporter [17]. ZIP proteins are thought to be mainly functioning as plasma membrane metal transporters, though some are localized to organellar membranes (e.g. [18]). *NcZNT1* is the orthologue of the *A*. *thaliana AtZIP4* gene, with 90% cDNA and 87% amino acid identity [19]. Heterologous expression in yeast showed it to mediate high-affinity Zn uptake and low-affinity Cd uptake, which would be in line with it residing in the plasma membrane [5]. The *AtZIP4* gene was not studied in great detail, but its expression is known to be strongly induced in roots and shoots under Zn deficient conditions [13, 20]. *NcZNT1* is expressed at higher levels in the Zn/Cd-adapted calamine *N*. *caerulescens* accessions Ganges and La Calamine than in the Ni-adapted serpentine accession Monte Prinzera, which suggests this gene is involved in the hyperaccumulation of Zn or Cd rather than Ni [21]. Transcription of *NcZNT1* is hardly affected by changes in Zn supply [17], contrary to transcription of *AtZIP4*, which is strongly induced upon Zn deficiency [22]. Only at very high Zn concentrations, the transcription of *NcZNT1* is somewhat reduced [5, 13]. This deregulation of *NcZNT1*, when compared to *AtZIP4*, seems to be part of the metal hyperaccumulation syndrome of *N*. *caerulescens*.

Recently Milner and colleagues [23] reported that NcZNT1 is able to transport Zn but not Cd, contradicting earlier reported observations from the same lab [5]. *A*. *thaliana* lines expressing *NcZNT1* were found to be sensitive to excess Zn, but not to Cd. However, for both studies, authors used what appears to be a 5’-truncated *NcZNT1* cDNA, which is unlikely to provide a wild-type protein *in planta*. Consequently, they also included part of the transcribed sequence in their analysis of the *NcZNT1* promoter. In the current study we have performed a detailed functional analysis of *NcZNT1*, based on the full-length wild-type cDNA as found in several accessions, as well as its proper promoter and 5’ UTR sequences. We first determined the response of *NcZNT1* transcription to changes in Zn supply. To understand the regulation pattern of *NcZNT1* and *AtZIP4* genes, we fused their full-length promoters and 5’ UTR sequences to *GUS* and *GFP* marker genes and studied their expression in *A*. *thaliana* and *N*. *caerulescens* under different metal exposure conditions. Furthermore, we examined the phenotype of *A*. *thaliana* lines expressing the full-length *NcZNT1* cDNA under control of the strong CaMV 35S promoter, including expression of other metal transporter genes in these lines, in an attempt to mimic the high expression of *NcZNT1* in a non-hyperaccumulator. We conclude that *NcZNT1* plays an important role in Zn and Cd tolerance and accumulation and is involved in establishing a high metal influx into the root vasculature, important for xylem-mediated translocation of metals to the shoot.

## Materials and Methods

### Isolation of *AtZIP4*, *NcZNT1* and orthologous promoters

Genomic DNA was extracted from flowers of *A*. *thaliana* (accession Columbia) and *N*. *caerulescens* (accession La Calamine), as described previously [24]. To amplify the sequence containing the *AtZIP4* promoter, a PCR reaction was performed on genomic DNA of *A*. *thaliana* using primers P5 and P15 (Table 1). To amplify the sequence containing the *NcZNT1* promoter, two forward primers (P1 and P2; Table 1) were designed on the gene upstream of *AtZIP4* (At1g10970) in *A*. *thaliana*. The reverse primer (P3; Table 1) was designed on the 5’ end of the *NcZNT1* cDNA (GenBank acc. No. AF275751) [17]. PCR fragments were cloned into pGEM-T-easy (Promega, Leiden, The Netherlands) and plasmids from several colonies for each fragment were sequenced to confirm their identity.

**Table 1:** Primers used for PCR amplifications. Restriction sites incorporated in the primers are underlined.

Primer	Sequence of oligonucleotides (5’-3’)	Purpose
P1	5’-ATCGGCGATGATCATGGGAA-3’	Forward primer for *NcZNT1* promoter isolation; designed on At1g10980
P2	5’-CCTCTTTTGGCCTCCATCGGAA-3’	Forward primer for *NcZNT1* promoter isolation; designed on At1g10980
P3	5’-TTATAAGATCAATCAATAATAACA-3’	Reverse primer for *NcZNT1* promoter isolation; designed on cDNA of *NcZNT1*
P4	5’- TAAAGTCGACGCCCAAATGGCGAGTGC -3’	Reverse primer on *NcZNT1* cDNA
P5	5’-GTAAGCTTTTGGAAAGTGAAGTGGATTG-3’	Forward primer for *AtZIP4* promoter isolation
P6	5’-CCAAGCTTAGATCTTGTCTGTTTTGTACTAACATGT-3’	Forward primer on *AtZIP4*
P7	5’-ATAAGCTTTCCACTGCAGAAACCGGTA-3’	Forward primer on *AtZIP4*
P8	5’-TGAAGCTTCCCATCTTACAAAGTTACCG TCCT-3’	Forward primer on *AtZIP4* promoter
P9	5’-TTAAGCTTCTTAAGCTACTCCTAATCATCCTTTTA-3’	Forward primer on *AtZIP4* promoter
P10	5’-TTAAGCTTTAGACTTGACTTAATCGGATTTTCT-3’	Forward primer on *AtZIP4* promoter
P11	5’-TGAAGCTTTTTGGAACAAATTGATTTTCTGTTT-3’	Forward primer on *AtZIP4* promoter
P12	5’-GAAAGCTTAATAACGCGAAAATGTCGACAT-3’	Forward primer on *AtZIP4* promoter
P13	5’-TTAAGCTTAGTATAGACAAGATTGGGAAGCTCT-3’	Forward primer on *AtZIP4* promoter
P14	5’-AGAAGCTTTCACTCTTTCTCCAAGTTGCCTCCT-3’	Forward primer on *AtZIP4* promoter
P15	5’-ATCGACGAAGACCATGGGAACAAGAGT-3’	Reverse primer for AtZIP4 promoter isolation
P16	5’-ATATCAAGCTTTCTGACTCTTTATCTGGCCTTTTA-3’	Forward primer on NcZNT1 promoter
P17	5’-TTAGAAGCTTAATACCTGATCTTGTCTG-3’	Forward primer on NcZNT1 promoter
P18	5’-GGAAGCTTTACGTAGCTGAAATGGAGGATGA-3’	Forward primer on NcZNT1 promoter
P19	5’-GGGAAGCTTGAAACAATCCAATCCTTAACC-3’	Forward primer on NcZNT1 promoter
P20	5’-TTAAGCTTCCGGTTTAGTGTGTTGAAGTTGTTAA-3’	Forward primer on NcZNT1 promoter
P21	5’-TTAAGCTTTCGTTTTTTTGTATTTCATGAACAA-3’	Forward primer on NcZNT1 promoter
P22	5’-AGAAGCTTCCATCATTACAATATTTTACTTGTCAAC-3’	Forward primer on NcZNT1 promoter
P23	5’-TTAAGCTTAAAAGGTGAAAAGAGAGAATAACG-3’	Forward primer on NcZNT1 promoter
P24	5’-AAATAAGCTTGTGTACAAGTGCCACGGAGC-3’	Forward primer on NcZNT1 promoter
P25	5’-ACAAGCTTTCGCTCGTCGATTCCTTCTTTTT-3’	Forward primer on NcZNT1 promoter
P26	5’-ATCGGCGATGACCATGGGAACAAAGA-3’	Reverse primer on NcZNT1 promoter
P27	5’-CCCAAGCTTACCCAAAAAAAGAGATCGAATT-3’	Forward primer on cDNA clone pAD-GAL4-2.1 vector with HinDIII
P28	5’-TAAAGTCGACGCCCAAATGGCGAGTGC-3’	Reverse primer for the NcZNT1 cDNA
P29	5’-TTCCCATGATCATCGCCGAT-3’	Forward primer designed on NcZNT1 cDNA
P30	5’-AAGCTTGCTGATAACTGTACTGGT-3’	Forward primer for PCR amplification of At-tubulin
P31	5’-GGTTTGGAACTCAGTGACATCA-3’	Reverse primer for PCR amplification of At-tubulin
P32	5’- CACCTTTGGAAAGTGAAGTG-3’	Forward primer on *AtZIP4* promoter
P33	5’- GGGAACAAGAGTTTATTC-3’	Reverse primer on *AtZIP4* promoter
P34	5’-CACCTCTGACTCTTTATCTGGCCT-3’	Forward primer on *NcZNT1* promoter
P35	5’-GGGAACAAAGAGTGTCTTCTTC-3’	Reverse primer on *NcZNT1* promoter

To isolate the *ZIP4/ZNT1* orthologous promoters from three related species, *Cochlearia pyrenaica* (accession La Calamine, Belgium), *Arabidopsis halleri* (accession Auby, France) and *Arabidopsis lyrata* (accession Unhost, Central Bohemia, Czech Republic) [25]—the latter two kindly provided by Dr. Pierre Saumitou-Laprade (CNRS, Lille, France)—genomic DNA was isolated and PCR amplified using the same primers (P1, P2, P3) as used for amplification of the *NcZNT1* promoter. PCR fragments were cloned into pGEM-T-easy (Promega, Leiden, The Netherlands) and inserts from several colonies for each fragment were sequenced to confirm their identity.

### Construction of binary plasmids

To generate a construct encoding a chimeric protein of enhanced (e)GFP fused to the C-terminus of NcZNT1 (*p35S*::*NcZNT1*:*eGFP*), a *NcZNT1* cDNA fragment of 1289 bp was amplified from the original cloned *NcZNT1* cDNA, using primers P27 and P28 (Table 1) and Pfu DNA polymerase (MBI Fermentas, St. Leon-Rot, Germany). The PCR fragment was digested with *Hin*DIII and *Sal*I and ligated into *Hin*DIII-*Sal*I digested binary vector pEZR(H)-LN (a kind gift from Gert-Jan de Boer, Carnegie Institution of Washington, USA) containing the *hpt* gene conferring plant hygromycin resistance. Cowpea protoplasts were prepared and transfected with the *p35S*::*NcZNT1*:*GFP* construct as described previously [26]. A *p35S*::*NcZNT1* construct was made by cloning the original 1.48-kb *NcZNT1* (accession La Calamine, Belgium) cDNA clone [17], upon restriction digestion with *Xba*I and *Hin*DIII, into the pGD121 vector [27] harbouring the *npt*II gene for plant selection on kanamycin resistance upon transformation.

A *pAtZIP4*::*GUS* construct, used for stable transformation of *A*. *thaliana*, was made by ligating a 1048-bp *A*. *thaliana* genomic DNA fragment, obtained by PCR using P5 and P15 (Table 1), upon digestion with *Hin*DIII and *Nco*I, at the ATG start codon of the *uidA* (*GUS*) gene in the *Hin*DIII—*Nco*I digested pCAMBIA1301 vector (http://www.cambia.org/daisy/bios/585.html) [28] replacing the CaMV 35S promoter. This vector contains the *hpt* gene for plant selection on hygromycin resistance. This construct was named F05. For the *pNcZNT1*::*GUS* construct, the *NcZNT1* promoter wa0-s cloned from *N*. *caerulescens* genomic DNA using primers P1 and P3, as described above, and the cloned fragment was re-amplified with primers P16 and P26 (Table 1) to create suitable restriction sites for cloning. Cloning was performed as described for the *AtZIP4* promoter. This construct was named F16.

In order to generate 5’ deletions of the *AtZIP4* and *NcZNT1* promoters, 10 forward primers containing appropriate *Hin*DIII and *Nco*I sites for cloning, were designed based on the promoter sequences of *AtZIP4* and *NcZNT1* (P5 through P14 for *AtZIP4* and P16 through P25 for *NcZNT1*) (Table 1). Each construct was named after the forward primers. In total, 22 constructs, 11 for *AtZIP4* and 11 for *NcZNT1*, were obtained following the same method as described for the F05 and F16 constructs. Two more constructs were made by digestion of the F05 and F16 vectors with *Sal*I, and subsequent self-ligation. These deletion constructs were named F15 in case of *NcZNT1* and F26 in case of *AtZIP4*. All constructs were verified by DNA sequencing. In addition, *pAtZIP4*::*eGFP* and *pNcZNT1*::*eGFP* constructs were developed based on the same pEZR(H)-LN binary vector used for making the *p35S*::*NcZNT1*:*eGFP* construct. The *pNcZNT1*::*eGFP* construct was made by replacing the CaMV35S promoter region of pEZR(H)-LN with the *NcZNT1 Hin*DIII-*Nco*I promoter fragment from F16. Similarly, the *pAtZIP4*::*eGFP* construct was made by replacing the CaMV35S promoter region with an *AtZIP4 Sac*I-*Nco*I promoter fragment from F05.

To generate a *pAtZIP4*::*GUS* construct suitable for hairy root transformation, the *AtZIP4* promoter was amplified from *A*. *thaliana* genomic DNA (accession Colombia) by using primers P32 and P33 (Table 1). Similarly, for a *pNcZNT1*::*GUS* construct suitable for hairy root formation, the *NcZNT1* promoter was amplified from *N*. *caerulescens* (accession La Calamine, Belgium) by using primers P34 and P35 (Table 1). The amplified fragments were cloned separately into the pENTR^TM^/D-TOPO® vector plasmid (Invitrogen^TM^, cat. K2400-20). These entry vectors were recombined into the binary destination vector, pKGWFS7-RR, by using the Gateway® LR Clonase^TM^ Enzyme Mix (Invitrogen^TM^, cat. 11791–019). pKGWFS7-RR contains the *uidH* gene encoding the GUS reporter protein and the promoter of the *A*. *thaliana UBIQUITIN10* (*AtUBQ10*) gene driving expression of the *DsRed* visible selection marker gene. DsRed expression can be used to identify transformed roots based on red fluorescence under a stereo microscope using a DsRed filter [29, 30]. The destination constructs were sequenced to confirm correct cloning of the *NcZNT1* and *AtZIP4* promoters.

### GUS staining

For qualitative GUS staining, the relevant plant parts were incubated at 37°C for 3 hours in a 50 mM sodium phosphate solution, buffered at pH 7.4, containing 1 mg/ml 5-bromo-4-chloro-3-indolyl b-D-glucuronide (X-Gluc). The stained plant parts were washed three times with 70% ethanol. Samples were observed using a Nikon Eclipse 80i microscope and images were captured using NIS Elements D3.1 software. A kinetic GUS assay was used for quantitative GUS analysis [31].

### GFP visualization

Transgenic roots identified by a Leica MZ FLIII Fluorescence Stereo Microscope were either or not immersed in 1 μg/mL propidium iodide for 1–5 minutes, and then washed with deionized water before imaging. Images were acquired with an inverted laser scanning confocal microscope (LSCM) system, Zeiss LSM 510 Meta (Carl Zeiss, Jena, Germany) or Zeiss LSM 5 PASCAL. The eGFP (green) signal was visualized with the excitation wavelength set at 488 nm and assembling emission signals between 505 to 530 nm. The signal for plant cell wall was visualized with the excitation wavelength set at 543 nm and assembling emission signals at 560 nm. A ×63 Plan Apochromate/ 1.4 oil DIC objective was used for examining transgenic *A*. *thaliana* roots, and EC Plan-Neofluar 20x or LD Plan-Neofluar 40x objectives were used for transgenic *N*. *caerulescens* roots. Digital images were processed using LSM 510 3.5 or LSM 5 Image Examiner software.

### Plant transformation and growth conditions

*A*. *thaliana* accession Columbia (Col) was stably transformed with *p35S*::*NcZNT1*, *pAtZIP4*::*GUS*, *pNcZNT1*::*GUS*, *pAtZIP4*::*eGFP*, *pNcZNT1*::*eGFP* and all *AtZIP4* and *NcZNT1* promoter deletion constructs using the *Agrobacterium tumefaciens*-mediated flower dipping transformation method [32]. Transformed seedlings (T_1_) were selected on ½ MS-agar plates [33] (no sugar, pH 5.8) supplemented with 50 mg L^-1^ kanamycin or 20 mg L^-1^ hygromycin (Duchefa Biochemie B.V., Haarlem, The Netherlands) at 24°C (16/8 hr, light/darkness). Fifty independently transformed plants were tested for *NcZNT1* expression by semi-quantitative RT-PCR (data not shown) and 10 high-expressing lines were propagated until stable homozygous T_3_ lines were obtained, which were used for experimentation. Plates were incubated in a climate-controlled growth cabinet (25°C 16/8 hr, light/darkness with illumination at a light intensity of 120 ***μ***mol m^-2^ s^-1^).

*N*. *caerulescens* roots were transformed with *pNcZNT1*::*GUS*, *pAtZIP4*::*GUS*, *pNcZNT1*::*eGFP* and *pAtZIP4*::*eGFP* constructs using a modified *Agrobacterium rhizogenes* mediated transformation method [34] to generate chimeric plants with transgenic hairy roots. Seeds of *N*. *caerulescens* were sterilized and geminated on ½ MS agar plates (no sugar, pH 5.8) at 24°C (16/8 hr light/darkness). Seven-day-old seedlings were cut above the hypocotyl-root boundary and roots were removed. A dot of *A*. *rhizogenes* (strain MSU440) containing a relevant construct was applied to the cut surface of each seedling and incubated for 5 day at 20/15°C (day/night, 12 hours light). The *A*. *rhizogenes*-inoculated seedlings were then transferred to ½ MS agar plates (no sugar, pH 5.8) containing 200 mg L^-1^ ticarcillin (Duchefa, Netherlands) at 24°C (16/8 hr, light/darkness) to remove *A*. *rhizogenes*. Non-transformed roots, which did not express DsRed or eGFP as determined using a Leica MZ FLIII Fluorescence Stereo Microscope, were cut off once every week until only transgenic roots were growing.

To determine the *NcZNT1* expression in response to various Zn treatments, seeds of wild-type *N*. *caerulescens* (La Calamine) were grown in modified half strength Hoagland’s nutrient solution containing 10 ***μ***M ZnSO_4_ [35]. After three weeks, the seedlings were supplied with different Zn concentrations for Zn deficiency (0.05 ***μ***M ZnSO_4_), Zn sufficiency (2 or 10 ***μ***M ZnSO_4_), or excess Zn (1000 μM ZnSO_4_). After another four weeks, shoots and roots were collected separately for gene expression analysis.

To determine the metal tolerance and accumulation of transgenic *p35S*::*NcZNT1 A*. *thaliana* lines, nine plants for each of three independent transgenic lines and one control *A*. *thaliana* wild type (WT) line were grown hydroponically in modified half strength Hoagland’s nutrient solution containing sufficient Zn (2 μM ZnSO_4_) or excess Zn (60 μM ZnSO_4_). For each treatment, the transgenic and control lines were grown in the same tray to avoid any effect of variation among the trays. Each tray contained about nine litres of nutrient solution. The plants were grown in a climate chamber (20/15°C day/night temperatures; 250 μmoles light m^-2^ s^-1^ at plant level during 12 h/day; 75% RH) for four to five weeks. In the first two weeks, plants were grown at sufficient Zn, and for the rest of the period at sufficient (control) or excess Zn. The nutrient solution was refreshed twice every week. Root and shoot tissues were harvested for metal concentration analysis. Each hydroponics experiment was replicated in time, while keeping all growth conditions the same.

To determine the response of *p35S*::*NcZNT1* transformed *A*. *thaliana* plants to Cd, the same transgenic lines were grown hydroponically on modified half strength Hoagland’s solution with sufficient Zn (2 μM ZnSO_4_) for two weeks and then transferred to the same medium but containing sufficient Zn (0 μM CdSO_4_ + 2 μM ZnSO_4_) and/or Cd (2 μM CdSO_4_ + 2 μM ZnSO_4_). The nutrient solution was refreshed every week. Plants were grown for four weeks.

For mineral concentration analysis roots and shoots were harvested individually. Roots were resorbed by dipping in 5 mM ice-cold Pb(NO_3_)_2_ for 30 min. Root and shoot samples were digested, and Zn, Cd, Fe, and Mn were measured spectrophotometrically as described by Assunção *et al*. [36].

For qualitative GUS analysis of transgenic *A*. *thaliana* containing *pAtZIP4*::*GUS* or *pNcZNT1*::*GUS* constructs, all homozygous lines were grown hydroponically on modified half strength Hoagland’s nutrient solution supplemented with either 2 μM ZnSO_4_ (sufficient Zn) or no Zn (Zn deficient). Based on the qualitative GUS expression results, three of these lines for each construct were selected for quantitative GUS analysis. These three lines (six seedlings per line) were initially grown on vertical ½ MS—1% agar plates for two weeks and then grown hydroponically on modified half strength Hoagland’s solution with sufficient Zn (2 μM ZnSO_4_) or Zn deficiency (no Zn added). Roots were collected every week for quantitative GUS analysis. The same was done for all other transformed *A*. *thaliana* plants with F05 through F26 constructs, except that roots were collected for quantitative GUS assay only once, after three weeks.

To compare *NcZNT1* and *AtZIP4* promoter activity in response to Zn treatments in *N*. *caerulescens* roots, chimeric *N*. *caerulescens* plants with transgenic hairy roots containing *pNcZNT1*::*GUS* or *pAtZIP4*::*GUS* constructs, were grown hydroponically on modified half strength Hoagland’s solution containing 0.05 μM ZnSO_4_ (Zn deficiency) or 10 μM ZnSO_4_ (sufficient Zn). The nutrient solution was refreshed every week. Plants were grown for four weeks. To examine the *NcZNT1* and *AtZIP4* promoter activity by confocal microscopy in *A*. *thaliana*, transgenic seedlings homozygous for *pNcZNT1*::*eGFP* or *pAtZIP4*::*eGFP* constructs, were grown in modified half strength Hoagland’s solution either containing no Zn (Zn deficiency) or 2 μM ZnSO_4_ (sufficient Zn) for three weeks. Similarly, chimeric *N*. *caerulescens* plants, with transgenic roots harbouring the *pNcZNT1*::*eGFP* or *pAtZIP4*::*eGFP* constructs, were transferred to half strength Hoagland’s solution containing either 0.05 μM ZnSO_4_ (Zn deficiency) or sufficient Zn supply (100 μM ZnSO_4_) for one week.

### RNA isolation and quantitative Reverse Transcriptase-PCR (qRT-PCR)

Total RNA was extracted with the RNeasy® Plant Mini kit (Qiagen). On-column DNase digestion was performed to remove any remaining genomic DNA. First strand cDNA was synthesized from 1 μg RNA using the iScriptTM cDNA Synthesis Kit (Bio-Rad). The *CLATHRIN* gene [37] was used as reference for normalization of transcription in *N*. *caerulescens* and the *UBIQUITIN-SPECIFIC PROTEASE6* gene (*UBP6*; At1g51710) for normalization of transcription in *A*. *thaliana*. Primers used for transcription analyses are shown in Table 2. Samples to which no reverse-transcriptase enzyme was added (NRT) were used as control for absence of genomic DNA.

**Table 2:** Primers used for quantitative reverse transcriptase PCR analyses.

Primer	Primer Sequence (5’-3’)
Forward primer for *NcZNT1*	GATCTTCGTCGATGTTCTTTGG
Reverse primer for *NcZNT1*	TGAGAGGTATGGCTACACCAGCAGC
Forward primer for *Clathrin*	AGCATACACTGCGTGCAAAG
Reverse primer for *Clathrin*	TCGCCTGTGTCACATATCTC
Forward primer for *AtUBP6*	GAAAGTGGATTACCCGCTG
Reverse primer for *AtUBP6*	CTCTAAGTTTCTGGCGAGGAG
Forward primer for *AtIRT1*	AAGCTTTGATCACGGTTGG
Reverse primer for *AtIRT1*	TTAGGTCCCATGAACTCCG
Forward primer for *AtIRT2*	ATGGCTACTACCAAGCTCGTC
Reverse primer for *AtIRT2*	CTAGACCGGACATCATAGCG
Forward primer for *AtFRO2*	CTTGGTCATCTCCGTGAGC
Reverse primer for *AtFRO2*	AAGATGTTGGAGATGGACGG
Forward primer for *AtBHLH100*	AAGTCAGAGGAAGGGGTTACA
Reverse primer for *AtBHLH100*	GATGCATAGAGTAAAAGAGTCGCT
Forward primer for *AtFRD3*	CGAGTTGCATCTCTTCTTCCT
Reverse primer for *AtFRD3*	TGATAACGGTCTCTCGAACA
Forward primer for *AtMTP1*	ACGGCCATGACCATCACAATC
Reverse primer for *AtMTP1*	TGCTTGTCCTCTCCATGACCA
Forward primer for *AtYSL3*	GAATTGAGAGACTAGTTTATTC
Reverse primer for *AtYSL3*	CGAGTTTTTACTTTTTGTGTAGCG
Forward primer for *AtNRAMP3*	ACAATGGGAGTCTCATTCGC
Reverse primer for *AtNRAMP3*	ATGCAACCCACAACTCCAAC
Forward primer for *AtHMA4*	ATGGCGTTACAAAACAAAG
Reverse primer for *AtHMA4*	GAGATTTGGTTTTACTGCTCTGAGC
Forward primer for *AtHMA3*	TTAAAGCTGGAGAAAGTATACCGA
Reverse primer for *AtHMA3*	GCTAGAGCTGTAGTTTTCACCT

Quantitative RT-PCR was performed using the iQTMSYBR® Green Supermix (Bio-Rad), including 12.5 μL of iQ SYBR Green Supermix, 5 pmol of forward and reverse primers, and 5 μl of 10 times diluted cDNAs (corresponding to 5 ng/μl RNA) in a total volume of 25 μl. PCR settings were 3 min at 95°C, followed by 40 cycles of 10 sec at 95°C and 1 min at 62°C. The fluorescence signal was detected with a CFX96TM Real-Time Detection System (Bio-Rad). Melting curves were analysed to confirm the absence of primer dimers and nonspecific products. Three biological repeats (each of three plants) per genotype or treatment and two technical repeats per biological repeat were used for the qRT-PCR analysis. The difference between technical repeats was less than 0.2 cycles. Relative transcript levels (RTLs) were calculated with the 2^-ΔΔCt^ method [38]. *NcZNT1* transcription of leaves under excess Zn (1000 μM ZnSO_4_) was used as the calibrator in *N*. *caerulescens* plants, which means its RTL value is set at 1. For wild-type and *p35S*::*NcZNT1* containing *A*. *thaliana* lines, transcription of target genes was normalized to the transcription in shoot of wild-type plants grown with sufficient Zn. *NcZNT1* transcription in the *p35S*::*NcZNT1* expressing *A*. *thaliana* line was normalized to its *AtHMA4* shoot transcription grown in sufficient Zn. The stability of reference genes was calculated by geNorm in qBasePLUS (Biogazelle) [39] and found to give geNorm M values lower than 0.5, which meant they were sufficiently stably transcribed to be used as reference [40]. A heat map used to present some qRT-PCR data was made using the online BAR HeatMapper Plus Tool (http://bbc.botany.utoronto.ca/ntools/cgi-bin/ntools_heatmapper_plus.cgi).

### Confirmation of the 5’ region of *NcZNT1* in three *N*. *caerulescens* accessions

To confirm the similarity of 5’ sequences of *NcZNT1* cDNAs of different *N*. *caerulescens* accessions with those previously published for the accession La Calamine [17], RNA was isolated from leaves of accessions La Calamine (LC), Prayon (PY), and Ganges (GA) using the RNeasy® Plant Mini kit (Qiagen). cDNA was synthesized using M-MLV Reverse Transcriptase (Invitrogen). *NcZNT1* cDNA fragments were PCR amplified using Pfu DNA polymerase (Fermentas) and forward (5’- GCTTTCTGCTCCTTGATCC -3’) and reverse (5’- CGATGAGAGGTATGGCTACA-3’) primers. The amplified fragments were cloned into the pGEM-T-easy vector (Promega) for DNA sequence analysis. *NcZNT1* gene fragments, covering the predicted exons [17], were amplified by using the same forward primer with reverse primer 5’-CTAAGCCCAAATGGCGA-3’ for PCR on genomic DNA of LC, PY, and GA, extracted using a modified nuclear extraction protocol [24]. The gene fragments were also cloned into pGEM-T-easy and DNA-sequenced. Resulting cDNA, genomic DNA and predicted protein sequences were compared using MultAlin software (http://multalin.toulouse.inra.fr/multalin).

### Statistical analyses

For statistical analyses, one-way and two-way ANOVA were performed, followed by Tukey’s test for multiple a posteriori comparison of individual means (SPSS Inc, Chicago, IL, USA). To obtain homogeneity of variances, data were log-transformed prior to analysis.

## Results

### *AtZIP4* and *ZNT1* cDNAs of three *N*. *caerulescens* accessions

**Fig 1:**
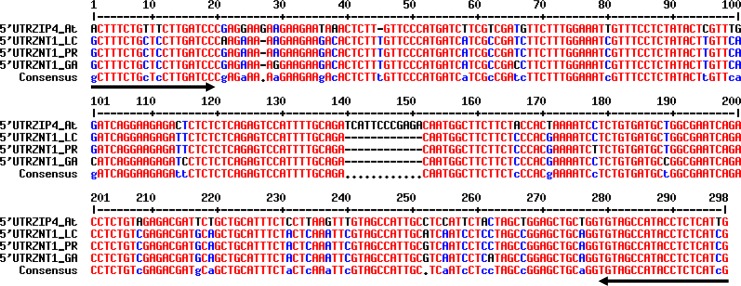
Comparison of 5’ ends of *NcZNT1* cDNAs isolated from *N*. *caerulescens* accessions La Calamine (LC), Prayon (PR), and Ganges (GA) to the 5’ end of the *AtZIP4* cDNA from *A*. *thaliana*. The first translational start codon (ATG) which is used as start codon in current study is at pos. 52–54. The second ATG, incorrectly interpreted as the translational start codon by [5, 23], is found at pos.154-156. Forward and reverse primers used for the amplification of these 5’ untranslated region (UTR) plus coding region cDNA fragments are shown as black arrows. GeneBank numbers of these sequences are: KU298434, KU298435 and KU298436 resp. for LC, PR and GA. The alignment was performed using MultAlin (http://multalin.toulouse.inra.fr/multalin).

When comparing the coding sequences (CDS) of *AtZIP4* (the At1g10970.1 gene model in TAIR; www.arabidopsis.org), *NcZNT1-LC* and *NcZNT1-PR* (deposited in GenBank (www.ncbi.nlm.nih.gov/nuccore/): AF275751.1, from *N*. *caerulescens* accession La Calamine, LC; AF133267.1, *N*. *caerulescens* accession Prayon, PR), we noticed that the *NcZNT1-PR* CDS appeared to be missing the first 5’ ~90 bp [23], when compared to the *NcZNT1-LC* and *AtZIP4* CDS, which removes 30 amino acids from the N-terminus of the predicted translated open reading frame (S1 Fig). To clarify if this difference was indeed due to a shorter transcript in the PR accession, we amplified the 5’ regions of *NcZNT1* cDNAs isolated from three *N*. *caerulescens* accessions, LC, PR, and Ganges (GA), using qRT-PCR. Sequence analysis showed that cDNAs from all three accessions contain the 5’ sequence as previously found in the *NcZNT1-LC* cDNA (GenBank acc. no. AF275751.1) (Fig 1). We thus conclude that the previously published Prayon *NcZNT1* CDS (GenBank AF133267.1) [5, 23], appears to represent an incomplete cDNA copy. The use of this cDNA fragment in expression studies would result in an N-terminally truncated protein compared to the original protein. To further clarify the gene structure, we also amplified the *NcZNT1* genomic regions from these three accessions, covering the predicted full length coding regions. The *NcZNT1* genomic DNAs are predicted to contain four exons and three introns in all these accessions, with an open reading frame of 1227 bp, which translates into 408 amino acids (S2 Fig) (GenBank acc. no. AAK69429.1) [17].

To confirm the presumed NcZNT1 protein localization at the plasma membrane [23], we transiently expressed a C-terminally GFP-tagged NcZNT1 fusion protein in cowpea protoplasts. The GFP signal was observed at the periphery of the cell, consistent with plasma membrane localization (S3 Fig). However, strong GFP signal was also observed in the cytoplasm, which is most likely due to the high expression of the construct driven by the CaMV35S promoter, causing improper targeting of the protein.

### *NcZNT1* transcription is up-regulated under Zn deficiency

The *NcZNT1* gene is known to be more or less constitutively transcribed in roots of *N*. *caerulescens*, almost irrespective of the Zn supply status [5, 13, 17], in contrast to its *A*. *thaliana* orthologue, *AtZIP4*, which transcription is strongly up-regulated under Zn deficiency. We determined *NcZNT1* transcription in roots and shoots in response to different Zn supplies, and though transcribed at Zn sufficient conditions, *NcZNT1* transcription was also induced by Zn deficiency, especially in shoots (~9-fold), and repressed by excess Zn treatment (S4A Fig).

### The *AtZIP4* and *NcZNT1* promoters are differentially regulated in *A*. *thaliana* and *N*. *caerulescens*

**Fig 2:**
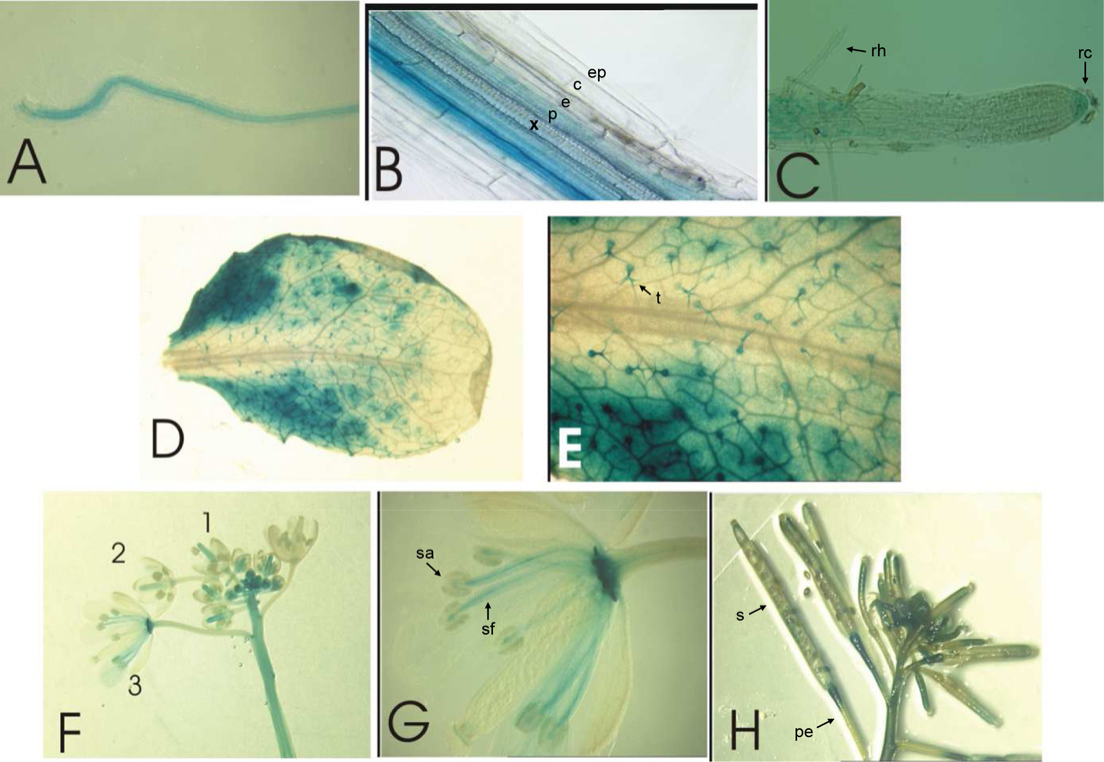
*pAtZIP4*::*GUS* expression in *A*. *thaliana* plants grown under Zn deficiency. GUS expression was analysed in transgenic plants grown hydroponically under Zn deficiency (no Zn added to half Hoagland’s nutrient solution). Expression was observed in: (A) a detached lateral root; (B) close-up of (A) showing GUS expression in endodermis and pericycle; (C) root tip and root hair zone; (D) leaf; (E) close up of (D) with expression in trichomes; (F) young inflorescence, indicating buds/flowers in increasing age (1, 2, 3); (G) close up of (F, 3) with expression in stamen filaments; (H) older inflorescence showing expression in siliques. Relevant tissues and organs are indicated: xylem (x), pericycle (p), endodermis (e), cortex (c), epidermis (ep), root hairs (rh), root cap (rc), trichomes (t), stamen filament (sf), stamen anther (sa), pedicel (pe), and silique (s).

**Fig 3:**
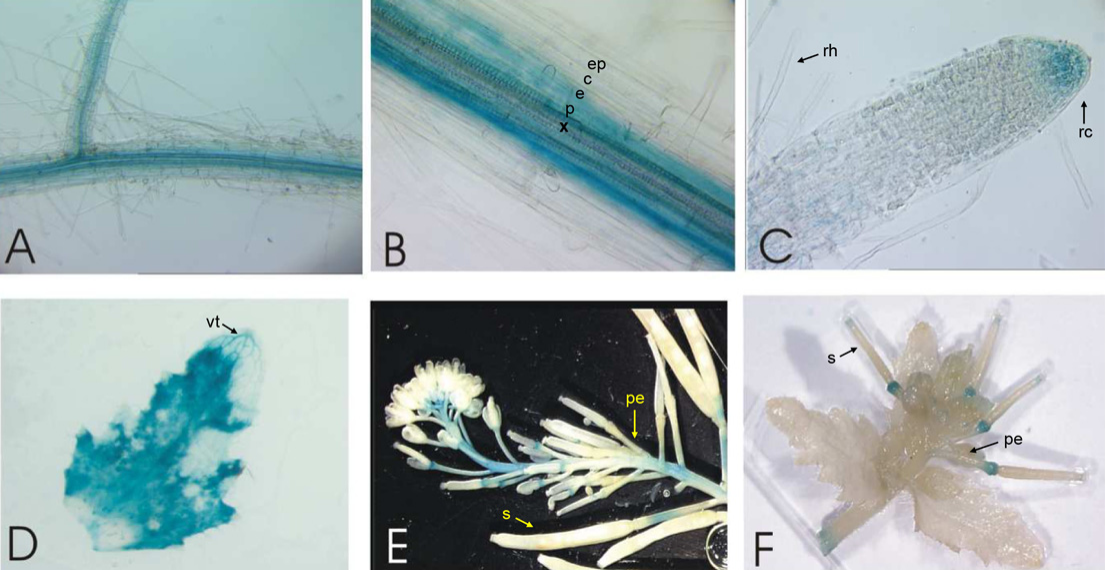
*pNcZNT1*::*GUS* expression in *A*. *thaliana* plants grown under Zn deficiency. GUS expression was analysed in transgenic plants grown hydroponically under Zn deficiency (no Zn added to half Hoagland’s nutrient solution). Expression was observed in: (A) a detached lateral root (B) close-up of (A) showing GUS expression in endodermis and pericycle; (C) root tip and root hair zone; (D) leaf; (E) and (F) inflorescence including siliques. Relevant tissues and organs are indicated: xylem (x), pericycle (p), endodermis (e), cortex (c), epidermis (ep), root hairs (rh), root cap (rc), trichomes (t), vascular tissue (vt), pedicels (pe), and silique(s).

In order to analyse the regulation of *AtZIP4* and *NcZNT1(-LC)*, we transformed promoter (p)::*GUS* constructs for both genes into *A*. *thaliana*. Plants from a homozygous *pAtZIP4*::*GUS-*transformed *A*. *thaliana* line were subsequently grown with deficient and sufficient Zn supply. GUS staining was observed only under Zn deficient conditions. The same was seen for a homozygous *pNcZNT1*::*GUS*-transformed *A*. *thaliana* line. GUS expression patterns were examined in more detail with several plants of these transgenic lines throughout their development. The roots of Zn deficient transgenic plants showed strong staining both in the tap root and lateral roots, specifically in the endodermis/pericycle and the root tip (Fig 2A–2C). A similar expression pattern was observed in the Zn-deficient roots of *pNcZNT1*::*GUS* transformed *A*. *thaliana* plants (Fig 3A–3C). *pAtZIP4*::*GUS* expression was also observed in leaves of Zn deficient plants, with most intense staining at the leaf edges (Fig 2D) and in the trichomes (Fig 2E). Similar expression patterns were also observed in *pNcZNT1*::*GUS*-transformed plants, although in these plants the trichome expression was not as obvious (Fig 3D). In Zn deficient *pAtZIP4*::*GUS* inflorescences, the highest GUS staining intensity was observed in the young buds attached to the main stem (Fig 2F) and in the pistils of older buds, in all developmental stages until just before opening. GUS was also expressed at the base of the open flower and the anther filaments (Fig 2G). After fertilization, the siliques express GUS at the distal ends; most prominently in young siliques and pedicels (Fig 2H). Again, very similar GUS expression was seen in Zn deficient *pNcZNT1*::*GUS* inflorescences (Fig 3E and 3F). The overall very similar GUS expression in *A*. *thaliana* plants carrying either the *AtZIP4* or the *NcZNT1* promoter driving GUS transcription, confirmed the orthology of both genes and showed that transcriptional regulation of these promoters is conserved.

**Fig 4:**
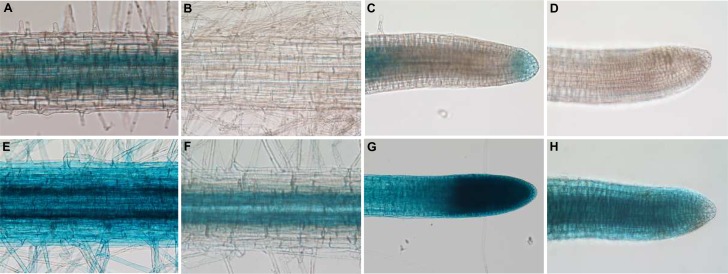
*pAtZIP4*::*GUS* and *pNcZNT1*::*GUS* expression in *N*. *caerulescens* roots. GUS expression was analysed in transgenic *N*. *caerulescens* roots grown hydroponically under Zn deficiency (0.05μM ZnSO_4_) (A, C, E, G) and Zn sufficiency (10 μM ZnSO_4_) (B, D, F, H). Expression was observed upon three-hour GUS staining of roots expressing *pAtZIP4*::*GUS* (A-D) or *pNcZNT1*::*GUS* (E-H). Images of mature roots (A, B, E, F) and root tips (C, D, G, H) are displayed.

Nevertheless, the endogenous *NcZNT1* transcription in *N*. *caerulescens* is higher than *AtZIP4* transcription in *A*. *thaliana* [13]. To confirm that both promoters reflect their endogenous activities, we also transformed both GUS constructs into *N*. *caerulescens*. In the absence of a reliable stable transformation system for *N*. *caerulescens*, we used *A*. *rhizogenes*-mediated root transformation [34], adapted for *N*. *caerulescens*. This results in chimeric plants, with a transgenic root system supporting a non-transgenic rosette. As in transgenic *A*. *thaliana*, both *AtZIP4* and *NcZNT1* promoters induced GUS expression under Zn deficiency, but the intensity of the staining differed considerably between constructs (Fig 4). The expression of *pAtZIP4*::*GUS* in *N*. *caerulescens* roots under Zn deficiency was restricted to root cap and stele, mainly endodermis and pericycle (Fig 4A and 4C). After 3 hours of GUS staining, *pAtZIP4*::*GUS* transgenic roots grown in sufficient Zn did not show any GUS expression (Fig 4B and 4D). Only upon overnight incubation, these roots showed a very weak GUS staining in the stele (data not shown). In the *pNcZNT1*::*GUS*-transformed *N*. *caerulescens* roots, the GUS expression was much higher than in *pAtZIP4*::*GUS* roots, with ubiquitous GUS staining throughout the root, including root tip, root hairs, epidermis, cortex and vasculature (Fig 4E and 4G), which was not observed for *pAtZIP4*::*GUS* roots (Fig 4A and 4C). The strongest expression appeared to be in the endodermis/pericycle. When supplied with sufficient Zn (10 μM ZnSO_4_), the *NcZNT1* promoter was less active than at Zn deficiency, without the high expression in the root tip and with the expression in mature roots limited to the stele (Fig 4F and 4H). Based on the repeatedly observed higher GUS expression in *pNcZNT1*::*GUS-* vs. *pAtZIP4*::*GUS*-transformed *N*. *caerulescens* roots, we conclude that in *N*. *caerulescens* the *pNcZNT1* promoter is more active than the *pAtZIP4* promoter, while the opposite (higher activity of the *pAtZIP4* promoter compared to the *pNcZNT1* promoter in *A*. *thaliana*) is not observed.

**Fig 5:**
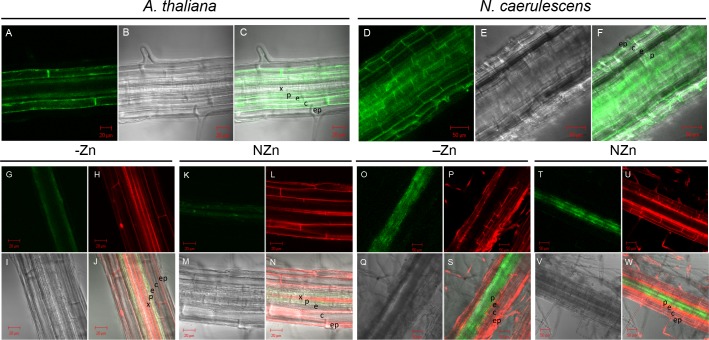
Confocal microscopy of GFP localization reflecting *AtZIP4* or *NcZNT1* promoter activity in *A*. *thaliana* and *N*. *caerulescens* roots. Transgenic *A*. *thaliana* plants (panels A-C, G-N) and transgenic *N*. *caerulescens* roots (panels (D-F, O-W) expressing *pAtZIP4*::*eGFP* (panels A-F) and *pNcZNT1*::*eGFP* (panels G-W) were grown hydroponically on half Hoagland’s nutrient solution to which no Zn was added (Zn deficiency, -Zn; panels A-C and G-J) or 2 μM ZnSO_4_ (normal Zn, NZn; panels K-N) (for *A*. *thaliana*), or to which 0.05 μM ZnSO_4_ was added (Zn deficiency, -Zn; panels D-F and O-S) or 100 μM ZnSO_4_ (normal Zn, NZn) (for *N*. *caerulescens*). Panels A, D, G, K, O and T show the GFP florescence image; panels H, L, P and U show fluorescence upon propidium iodide staining; panels B, E, I, M, Q and V show the differential interference contrast (DIC) images; and panels C, F, J, N, S and W the merged images of each set. Root cell types are indicated with letters; x (xylem), p (pericycle), e (endodermis), c (cortex) and ep (epidermis). Scale bars are indicated.

While the GUS assay is a quick and convenient way to detect patterns of promoter activity, there is also the issue of diffusion of the enzyme, especially at high GUS expression, which disturbs precise localization of promoter activity. Therefore we also cloned the promoters upstream of the enhanced Green Fluorescent Protein (eGFP) gene, adapted for expression in planta, and used these constructs for *A*. *tumefaciens* transformation of *A*. *thaliana* and *A*. *rhizogenes* transformation of *N*. *caerulescens*, upon which roots were examined by confocal microscopy. This showed that the *pAtZIP4*::*eGFP* construct directs GFP expression to the cortex, endodermis and pericycle in Zn deficient *A*. *thaliana* roots (Fig 5A–5C). Expression was not observed in *A*. *thaliana* roots supplied with sufficient Zn. In the Zn deficient *N*. *caerulescens* roots the expression appears to be slightly higher than in *A*. *thaliana* and the expression is not confined to cortex, endodermis and pericycle, but also visible further inside the stele, as well as in the epidermis (Fig 5D–5F). Similar to *A*. *thaliana*, GFP expression was never observed in *pAtZIP4*::*eGFP N*. *caerulescens* roots supplied with sufficient Zn.

The GFP expression in the *pNcZNT1*::*eGFP A*. *thaliana* roots under Zn deficiency (Fig 5G–5J) was comparable to expression of *pAtZIP4*::*eGFP* (Fig 5A–5C), although expression was confined to the endodermis/pericycle and was not obvious in the cortex. Also plants grown at sufficient Zn supply showed GPF expression in the endodermis/pericycle (Fig 5K–5N). The GFP expression in *pNcZNT1*::*eGFP* transformed *N*. *caerulescens* roots (Fig 5O–5W) was mostly in the pericycle, and further inside the stele, but at much lower levels in the endodermis. There was not much difference when comparing Zn deficient and Zn sufficient roots (Fig 5O–5W).

### *pAtZIP4*::*GUS* and *pNcZNT1*::*GUS* mediated GUS expression in *A*. *thaliana* peaks after three weeks Zn deficiency

The quantitative GUS expression response of the *AtZIP4* and *NcZNT1* promoters to Zn deficiency was determined for *pAtZIP4*::*GUS-* and *pNcZNT1*::*GUS*-expressing *A*. *thaliana* plants grown hydroponically in half Hoagland’s solution containing different Zn concentrations (0, 0.1, 0.2, 0.5, 1, 2 μM ZnSO_4_) (S5 Fig). Only the two lowest Zn concentrations, no Zn added and 0.1 μM ZnSO_4_, induced GUS expression in roots, which peaked after three weeks of exposure. A very similar time course of GUS expression was observed for *pNcZNT1*::*GUS* and *pAtZIP4*::*GUS* plants, though in the former, induction of GUS expression started a week later.

### Conserved regulatory *cis* elements in *AtZIP4* and *NcZNT1* promoters are essential for their Zn deficiency responsive activity

**Fig 6:**
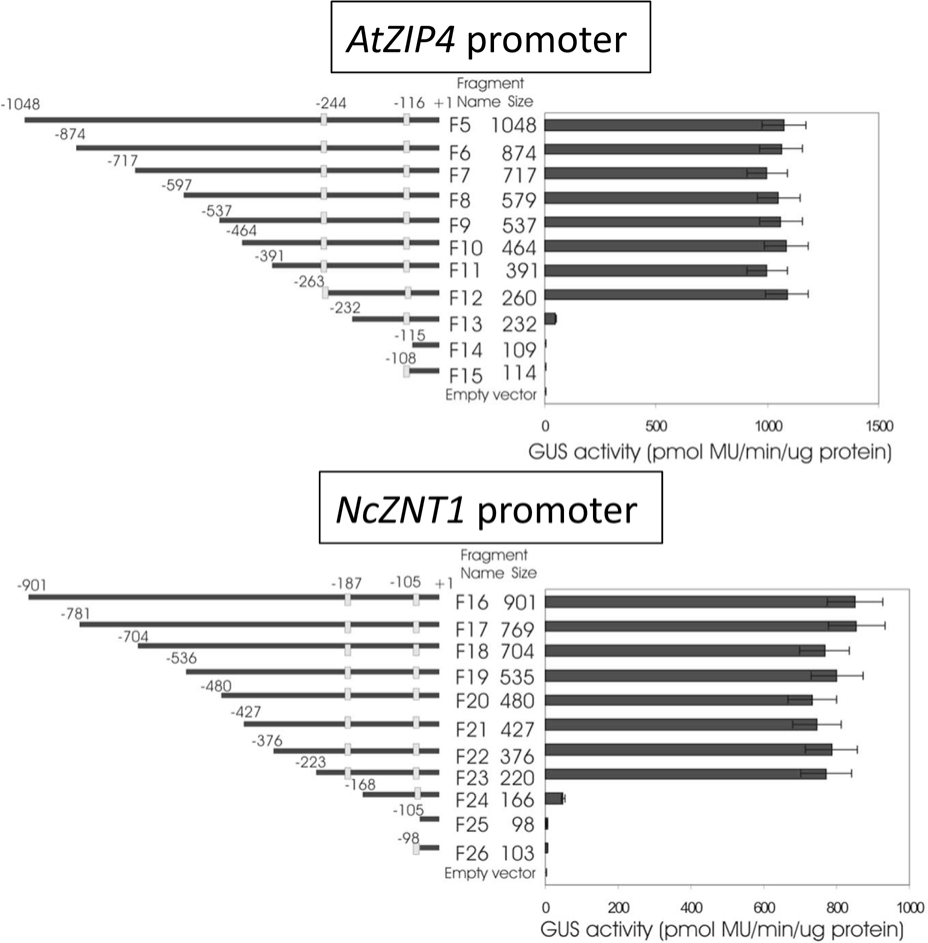
The effect of 5’ deletions of the *AtZIP4* and *NcZNT1* promoters on GUS expression. The GUS activity (pmole/min/μg protein) was tested in roots of transgenic (A) *pAtZIP4*::*GUS* and (B) *pNcZNT1*::*GUS A*. *thaliana* plants exposed to 0 μM Zn.

A 5’ deletion analysis of the *AtZIP4* and *NcZNT1* promoters was performed to identify *cis* elements in these two promoters involved in the regulation of these genes. Quantitative GUS expression was used as marker for promoter activity. Almost 0.8 kbp can be deleted from the 5’ end of the *AtZIP4* promoter and almost 0.7 kbp of the *NcZNT1* promoter without significantly affecting promoter-fragment-driven GUS expression (Fig 6). Only when a previously identified palindromic *cis* element, known as a Zn Deficiency Responsive Element (ZDRE) [22] was deleted, from either promoter, the GUS activity was significantly reduced. Removal of the second ZDRE reduced GUS expression to background levels. Except for these two *cis* elements, there appeared to be no other relevant regulatory elements in these promoters.

### Orthologous *ZIP4* promoters from *Arabidopsis lyrata*, *Arabidopsis halleri* and *Cochlearia pyrenaica* share the conserved ZDREs found in the *AtZIP4* and *NcZNT1* promoters

Since only the two ZDREs appeared to be relevant for *AtZIP4* or *NcZNT1* promoter activity in response to Zn deficiency, we were interested to determine if only these sequences were conserved when comparing promoters of *ZIP4* orthologues of other Brassicaceae. PCR amplification of *ZIP4* promoter fragments from *A*. *lyrata*, *A*. *halleri* and *C*. *pyrenaica* yielded single bands of between 0.6 to 1.2 kbp, except for *A*. *halleri* for which two bands were obtained. All fragments were cloned and sequenced (Table 3). The difference in length between both *A*. *halleri* fragments was caused by a 154-bp insertion/deletion. The common sequence of both fragments was nearly identical, suggesting that these two fragments either represent two gene copies or two alleles in this strictly out-crossing species. The sequence comparison of all *ZIP4* orthologous promoters showed only a low level of sequence conservation (S6 Fig). Interestingly, the two ZDREs were conserved in the *ZIP4* orthologous promoters from all five species, all located close to the predicted ATG start codon of the gene and all with similar distance between both ZDREs.

**Table 3:** DNA fragment characteristics of *ZIP4*-orthologous promoters isolated from *Noccaea caerulescens*, *Cochlearia pyrenaica*, *Arabidopsis halleri* and *Arabidopsis lyrata*. Sequence lengths are provided. For *A*. *halleri* two fragments were amplified, differing in size due to a 154-bp InDel. Positions of identified sequence boxes, corresponding to two Zinc Deficiency Responsive Elements (ZDRE-1 and -2; consensus sequences indicated) and a predicted TATA box, are indicated relative to the predicted transcription start.

Species name	promoter fragment	ZDRE-1 (bp)	ZDRE-2 (bp)	Predicted
	length (bp)	[5’-ATGTCGACAT-3’]	[5’-ATGTCGACAC3’]	TATA box (bp)
***A*. *thaliana***	1048	-246 to -236	-118 to -108	-59 to -55
***N*. *caerulescens***	902	-189 to -179	-107 to -97	-70 to -66
***C*. *pyrenaica***	571	-235 to -225	-116 to -106	-59 to -55
***A*. *halleri(long)***	905	-221 to -211	-115 to -105	-58 to -54
***A*. *halleri(short)***	746	-221 to -211	-115 to -105	-58 to -54
***A*. *lyrata***	1189	-235 to -225	-116 to -106	-59 to -55

### High heterologous expression of *NcZNT1* confers early flowering, increased Zn tolerance and Zn accumulation to *A*. *thaliana*

**Fig 7:**
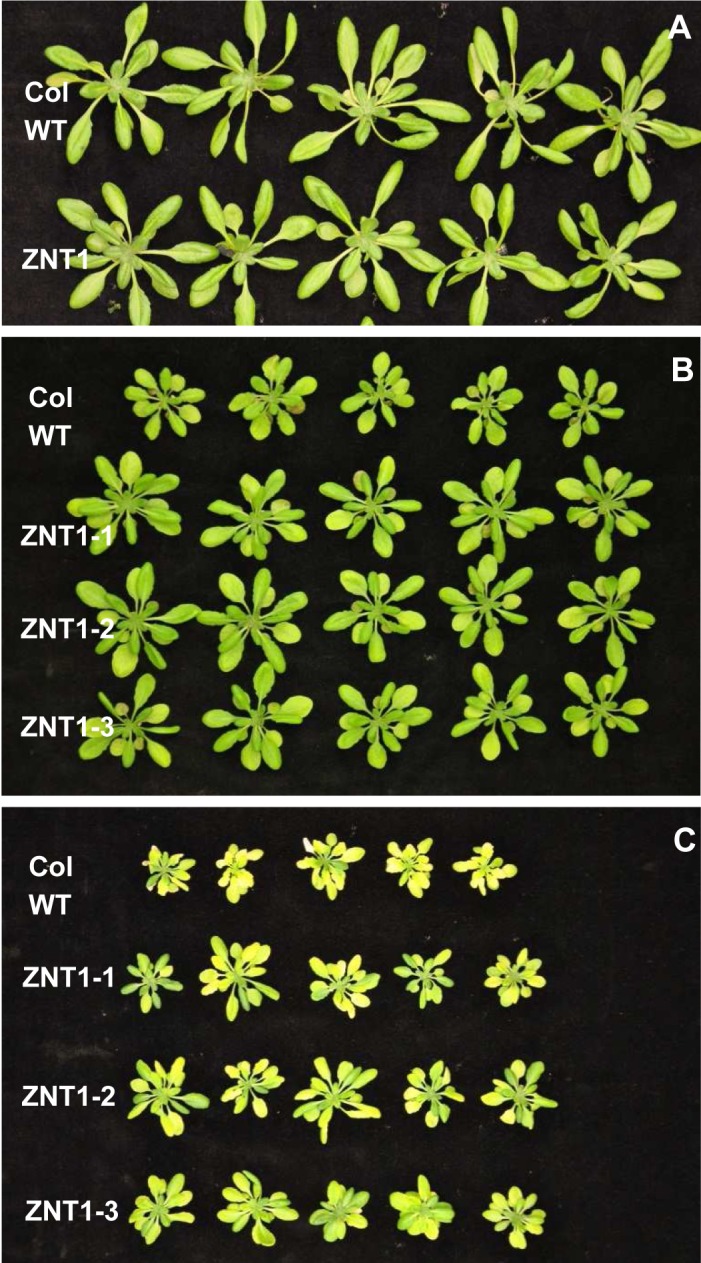
Expression of NcZNT1 in *A*. *thaliana* confers tolerance to excess Zn and Cd exposure. Three independently transformed lines expressing a *35S*::*NcZNT1* construct (NcZNT1-1, NcZNT1-2, NcZNT1-3) and Col wild-type plants (Col-WT) were grown hydroponically for four weeks, first one week on half Hoagland’s media containing 2 μM ZnSO_4_, thereafter on media containing 2 μM ZnSO_4_ (A), 60 μM ZnSO_4_ (B) and 2 μM CdSO_4_ + 2 μM ZnSO_4_ (C).

**Fig 8:**
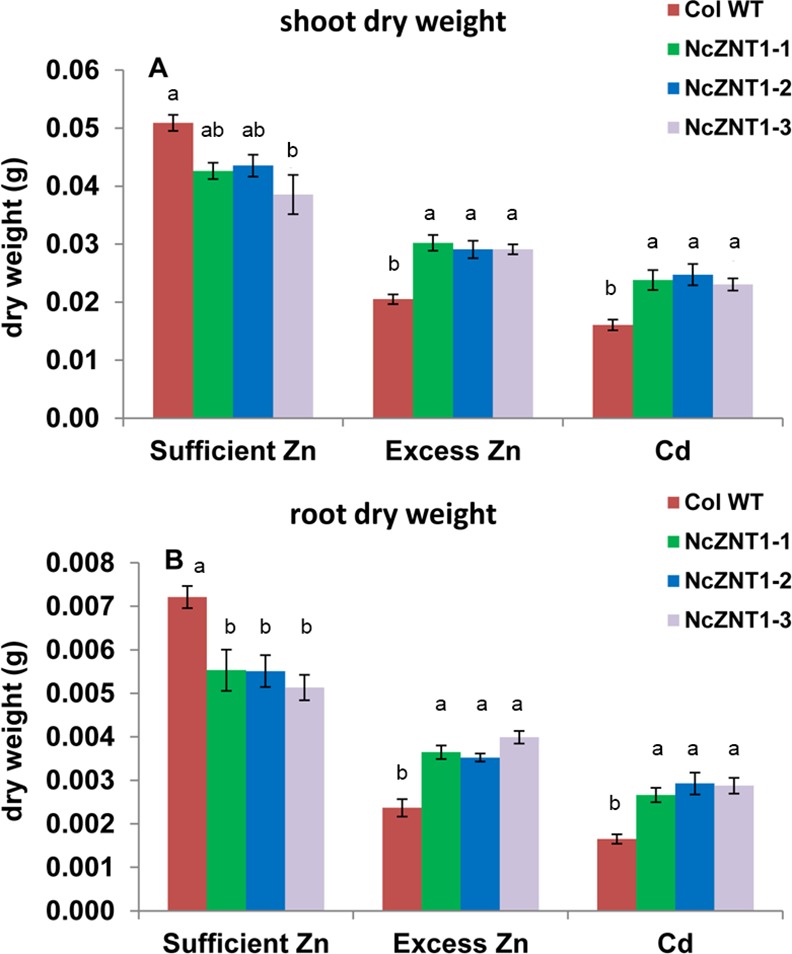
Tolerance of NcZNT1-expressing *A*. *thaliana* to excess Zn and Cd exposure corresponds to increased dry biomass. Shoot (A) and root dry weights (B) are shown of three independently transformed *A*. *thaliana* lines expressing a *35S*::*NcZNT1* construct (NcZNT1-1, NcZNT1-2, NcZNT1-3) and Col wild-type (WT) plants grown hydroponically for four weeks, first one week on half Hoagland’s media containing 2 μM ZnSO_4_, thereafter on media containing 2 μM ZnSO_4_ (sufficient Zn), 60 μM ZnSO_4_ (excess Zn) and 2 μM CdSO_4_ + 2 μM ZnSO_4_ (Cd). Error bars represent the standard errors of the mean. Different letters indicate significant differences (p<0.05) among plant types within treatments (transgenic lines and wild-type).

**Fig 9:**
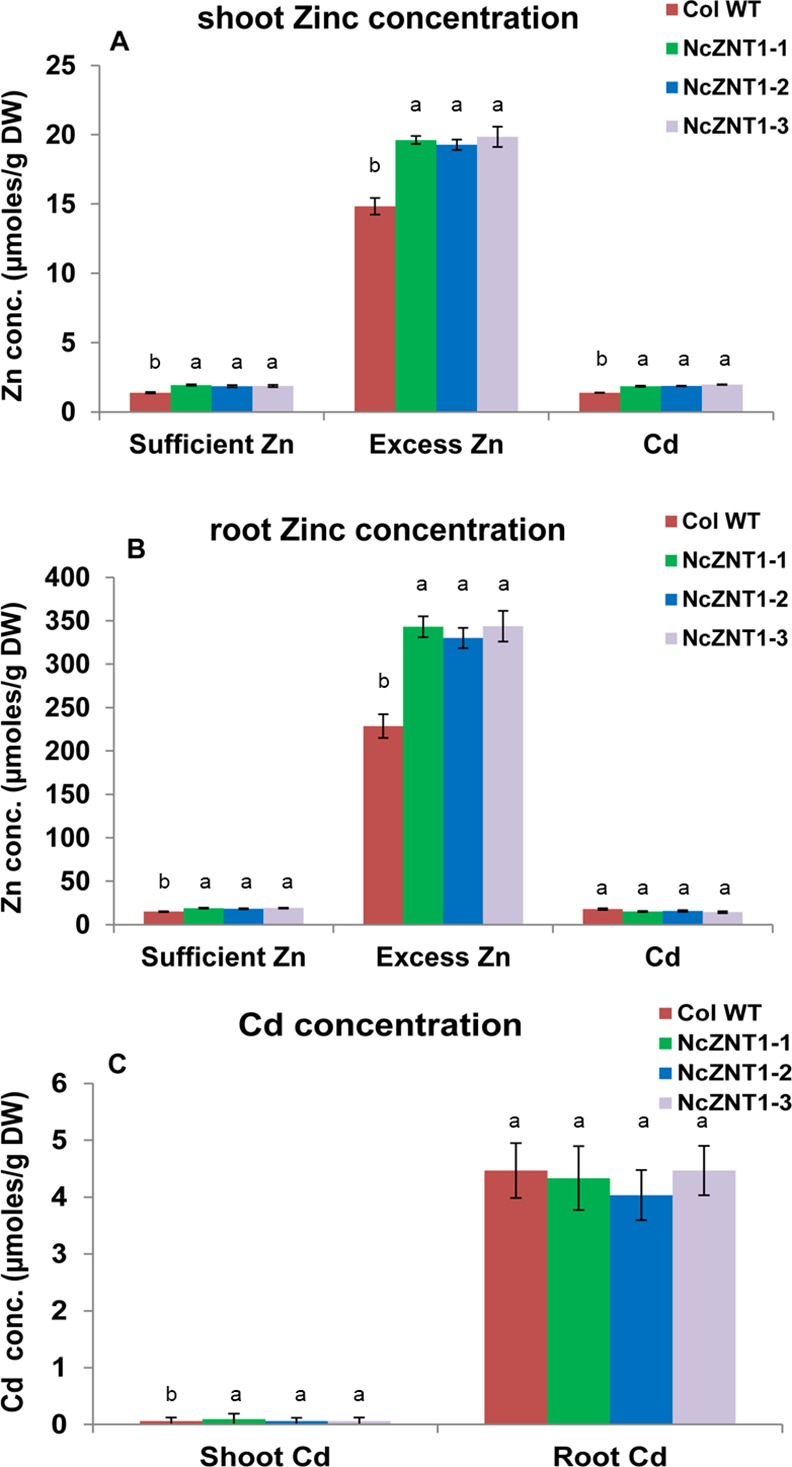
Expression of NcZNT1 enhances the Zn and Cd concentrations of *A*. *thaliana*. Shoot (A) and root Zn (B) and Cd (C) concentrations (conc., in μmoles per gram dry weight) are shown of three independently transformed *A*. *thaliana* lines expressing a *35S*::*NcZNT1* construct (NcZNT1-1, NcZNT1-2, NcZNT1-3) and Col wild-type (WT) plants grown hydroponically for four weeks, first one week on half Hoagland’s media containing 2 μM ZnSO_4_, thereafter on media containing 2 μM ZnSO_4_ (sufficient Zn), 60 μM ZnSO_4_ (excess Zn) and 2 μM CdSO_4_ + 2 μM ZnSO_4_ (Cd). Error bars represent the standard errors of the mean (n = 4). Different letters indicate significant differences (p<0.05) among plant types within treatments (transgenic lines and wild-type).

To investigate if high expression of *NcZNT1* would be sufficient to increase Zn uptake in plants, we expressed it in transgenic *A*. *thaliana* using the strong double CaMV 35S promoter to drive transcription (*p35S*::*NcZNT1*) (S4B Fig). When homozygous *p35S*::*NcZNT1 A*. *thaliana* lines were grown on modified half Hoagland’s nutrient solution containing sufficient Zn (2 μM ZnSO_4_), they all flowered two to four days earlier than wild-type Col-0 (WT) plants (S7 Fig). No additional abnormal visible phenotype was discerned at this Zn supply. When the same lines were grown on Zn deficient medium (no Zn added), they appeared to be more sensitive to zinc deficiency than the WT plants (S8A Fig). The experiment was repeated to determine the zinc concentration in the plants, but this time the plants were grown on Zn deficient medium with a very low zinc concentration (0.05 μM ZnSO_4_) to allow some Zn uptake. The Zn concentration in shoots and roots of the *p35S*::*NcZNT1* plants was significantly higher than in the WT plants (S8B Fig). Again, no abnormal morphological phenotypes were observed for the transgenics grown at sufficient Zn (Fig 7A), however, root dry weights of the transgenic lines were lower than those of the WT line (Fig 8B). The reverse was seen when plants were exposed to excess Zn. This high Zn supply affected growth of the WT line much more than that of the transgenic lines, leading to significantly higher shoot and root dry weights of the transgenic lines (Fig 7B; Fig 8A and 8B). WT plants displayed purple pigmentation of the older leaves when grown at excess Zn, which was not observed for transgenic plants. Like plants grown at Zn deficiency, the *p35S*::*NcZNT1* plants grown on sufficient Zn, and particularly when grown on excess Zn, had a markedly higher Zn concentration in shoots and roots than WT plants (Fig 9A and 9B). They also contained higher concentrations of Mn, but not Fe, in shoots compared to WT. Roots of transgenic and WT lines were similar with regards to Fe and Mn concentrations (S9A–S9D Fig).

### *NcZNT1* expressing *A*. *thaliana* lines showed enhanced Cd tolerance and accumulation

Since NcZNT1 has been reported to transport Cd [5], we exposed the *p35S*::*NcZNT1* plants to the same nutrient solution used for sufficient Zn supply, supplemented with 2 μM CdSO_4_. As we observed for the excess Zn response, the transgenic plants were more tolerant to Cd exposure than WT, with larger rosette size and leaves with less chlorosis (Fig 7C). Also the shoot and root dry weights of the transgenic lines were significantly higher than those of the WT line (Fig 8A and 8B), as well as the shoot Zn and Cd concentrations (Fig 9C). However, the shoot Cd concentrations remained much lower than the root Cd concentrations (Fig 9C), indicating that *A*. *thaliana* manages to retain Cd in roots at this level of Cd supply. Only shoot Fe concentrations decreased in the transgenic lines upon Cd exposure while the Mn and root Fe concentrations were comparable to those in WT (S7 Fig).

**Fig 10:**
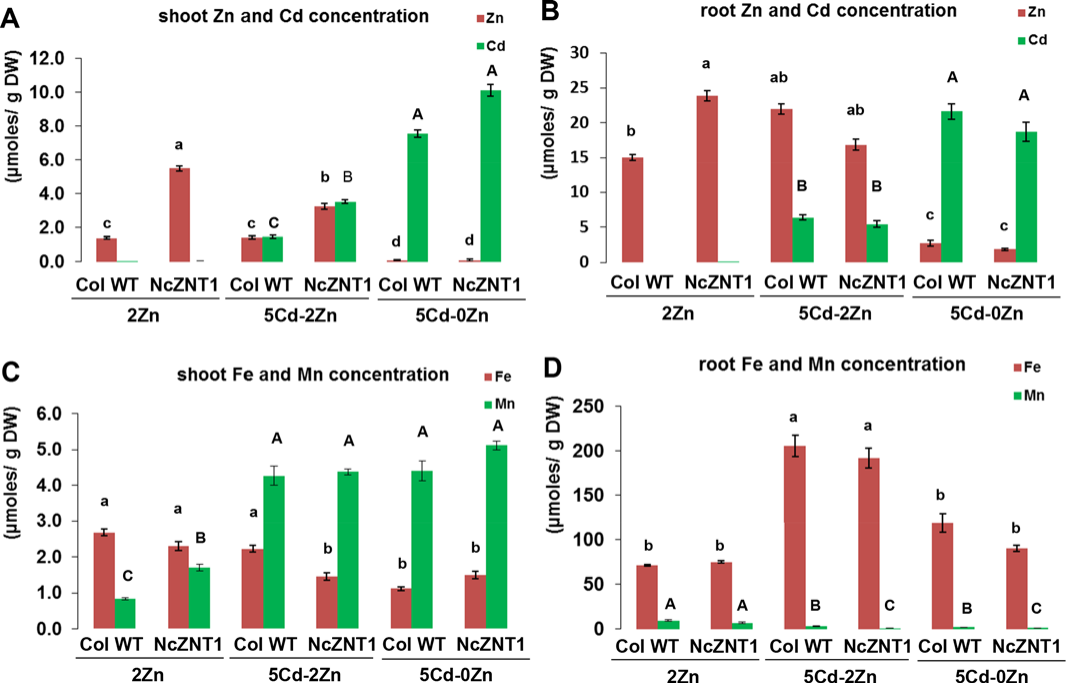
Zn, Cd, Fe, and Mn concentrations of *A*. *thaliana* are affected by expression of NcZNT1. Shoot (A, C) and root (B, D) concentrations (conc.) of Zn and Cd (A, B) and Fe and Mn (C, D) (in μmoles per gram dry weight) are shown of three independently transformed *A*. *thaliana* lines expressing a *35S*::*NcZNT1* construct (NcZNT1-1, NcZNT1-2, NcZNT1-3) and Col wild-type (WT) plants grown hydroponically for four weeks, first one week on half Hoagland’s media containing 2 μM ZnSO_4_, thereafter on media containing 2 μM ZnSO_4_, no Cd (0 Cd – 2 Zn), 5 μM CdSO_4_ + 2 μM ZnSO_4_ (5 Cd – 2 Zn) and 5 μM CdSO_4_, no Zn (5 Cd – 0 Zn). Error bars represent the standard errors of the mean (n = 4). Different letters (small case for Zn and Fe; capitals for Cd and Mn) indicate significant differences (p<0.05) among plant types (transgenic lines and wild-type) and treatments.

The differences between transgenic and WT plants for the concentrations of metals of which exposure levels were not altered, suggests there may be competition between metals for entering plant cells. To study this further, one representative *p35S*::*NcZNT1* line was compared with WT by growing plants on half Hoagland’s media supplemented with either 2 μM ZnSO_4_, no Cd (2 Zn 0 Cd); 5 μM CdSO_4_ + 2 μM ZnSO_4_ (2 Zn 5 Cd) or 5 μM CdSO_4_, no Zn (0 Zn 5 Cd), while keeping the other mineral concentrations constant. At this much higher Cd exposure level, there was no longer a morphological phenotypic difference between transgenic and WT plants. Both lines were equally affected, as displayed by severe chlorosis and stunted growth (data not shown). Nevertheless, the transgenic plants contained a significantly higher shoot (but not root) Cd concentration compared to WT (Fig 10A and 10B). Like in the previous experiments, shoot Zn concentrations were higher in transgenic than WT plants, except for plants grown in the absence of Zn, which showed equally low shoot Zn concentrations. WT plants showed little evidence for competition of Cd with Zn or Fe and Mn, the other two metals measured (Fig 10C and 10D). On the contrary, exposure to 2 Zn 5 Cd enhanced shoot Mn concentrations and root Fe concentrations. There appeared to be some competition between Cd and Zn regarding root to shoot metal transport involving NcZNT1, as the presence of Cd lowered the Zn concentration of transgenic shoots compared to only Zn exposure. In the absence of Zn, shoot and root Cd concentrations reached high levels, much higher than when Zn was present (Fig 10A and 10B) (p<0.001 and p<0.05 for plant type x treatment interactions in shoot and root, respectively).

### Expression of *NcZNT1* alters the expression of other metal homeostasis genes in *A*. *thaliana*

**Fig 11:**
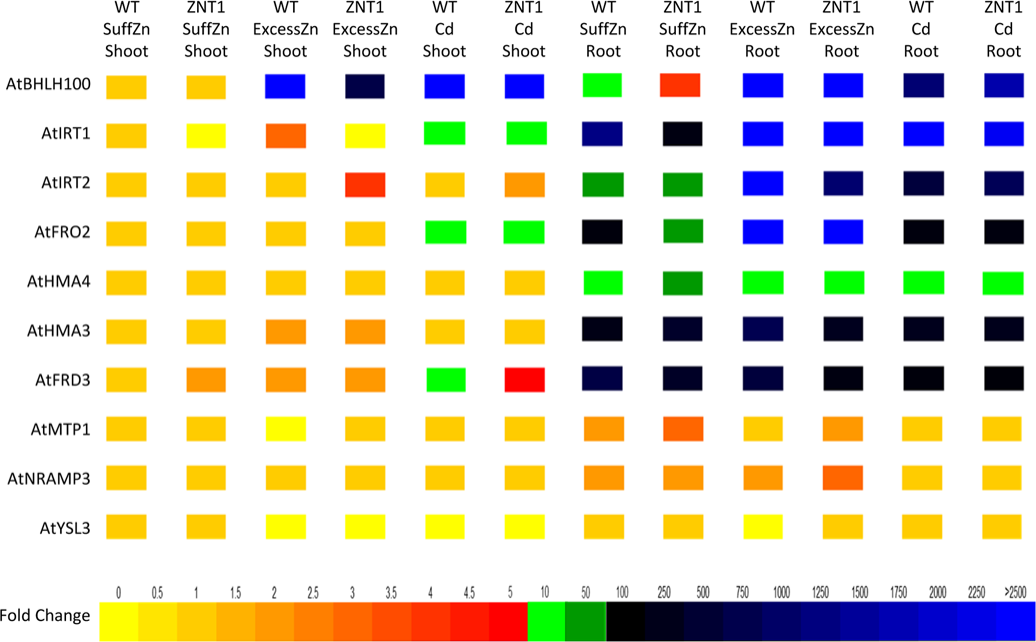
Expression of NcZNT1 affects the transcription of Zn and Fe homeostasis genes of *A*. *thaliana*. Relative transcription analysis of genes involved in Zn and Fe homeostasis in shoots (Shoot) and roots (Root) of a transformed *A*. *thaliana* line expressing a *35S*::*NcZNT1* construct (ZNT1) and Col wild-type plants (WT) in response to two weeks exposure to 2 μM ZnSO_4_ (SuffZn), 60 μM ZnSO_4_ (ExcessZn) and 2 μM CdSO_4_ + 2 μM ZnSO_4_ (ExcessCd). The relative transcription of genes in WT plants exposed to 2 μM ZnSO_4_ was set to 1 (first column) and fold-changes of transcription are indicated as a heat map, with the corresponding legend included at the bottom. Transcription of *AtUBP6* (At1g51710) was used as reference to normalize the quantitative reverse transcriptase PCRs.

Considering that expression of *NcZNT1* alters the *A*. *thaliana* Zn and Cd accumulation and tolerance characteristics, we determined the expression of the Zn and Fe homeostasis genes *AtBHLH100*, *AtIRT1*, *AtIRT2*, *AtFRO2* (involved in Fe uptake); *AtNRAMP3* (involved in Fe remobilization); *AtHMA4* (involved in Zn/Cd translocation); *AtYSL3*, *AFRD3* (involved in Zn/Fe translocation); and *AtMTP1*, *AtHMA3* (involved in Zn/Cd vacuolar storage) upon exposure of WT and transgenic plants to sufficient Zn (2 μM ZnSO_4_), excess Zn (60 μM ZnSO_4_) or Cd (2 μM CdSO_4_ + 2 μM ZnSO_4_). The genes involved in Fe uptake, *AtIRT1*, *AtIRT2* and *AtFRO2*, are mainly expressed in roots, whereas the transcription factor gene *AtbHLH100* is expressed both in roots and shoots. Transcription levels of all of these genes go up upon excess Zn and Cd exposure (Fig 11; S1 Table), indicating that this induces a Fe deficiency response. However, in the *p35S*::*NcZNT1* transgenic line, the transcription of *AtbHLH100*, and its targets *AtIRT1*, *AtIRT2* and *AtFRO2*, as well as that of *AtFRD3*, is less induced by excess Zn than in WT plants. Cd had an enhancing effect on transcription of *AtbHLH100* and *AtIRT2*. Similar effects were seen for *AtIRT1*, *AtIRT2* and *AtFRO2* transcription in shoot, though the biological relevance of this is doubtful considering the much lower transcription of these genes in shoots. *AtMTP1* is transcribed in roots and shoots, with higher transcription in transgenic plants than in WT upon excess Zn and Cd. Transcription of *AtYSL3* and *AtNRAMP3* was only increased in roots of transgenic plants upon excess Zn. *AtHMA3* was mainly transcribed in roots, with higher transcription in WT than in transgenic plants upon excess Zn. Also *AtHMA4* was transcribed at higher levels in roots than in shoots, but the difference was much less than for *AtHMA3*. While at sufficient Zn supply both genes were higher transcribed in transgenic roots compared to WT ones, it was the reverse upon excess Zn exposure.

## Discussion

We found *NcZNT1* to be transcribed in both shoots and roots of *N*. *caerulescens* under Zn deficiency and sufficiency, with reduced transcription under Zn excess (S4 Fig). An NcZNT1:eGFP fusion protein localizes to the plasma membrane when expressed in cowpea protoplasts (S3 Fig). These observations are in line with previous reports [5, 17, 23] that NcZNT1 is a Zn transporter involved in cellular Zn influx. Since *NcZNT1* is transcribed in both roots and shoots, and predominantly in the pericycle and the endodermis, it is more likely to have a function in making Zn available for xylem transport through the plant than to be solely involved in Zn uptake from the soil.

Using primers designed on the gene located upstream of *AtZIP4* in *A*. *thaliana*, we were able to PCR-amplify the *NcZNT1* promoter. The micro co-linearity between both species confirms that both genes originate from the same gene in their most recent common ancestor. The *AtZIP4* and *NcZNT1* promoter-*GUS* and -*GFP* marker gene fusions allowed us to study their gene expression in great detail (Figs 2–5). The *AtZIP4* gene is not transcribed under sufficient Zn supply, but only in response to Zn deficiency in *A*. *thaliana* [13, 20], which is also what we found for *AtZIP4* expression in *N*. *caerulescens*. This in contrast to *NcZNT1* promoter activity in *N*. *caerulescens*, which is expressed under sufficient Zn supply, and which is also more active than the *AtZIP4* promoter in either species (Figs 4 and 5). The GFP expression analysis basically confirmed the root GUS expression analysis, but allowed a better localization of the promoter activity. The *AtZIP4* and *NcZNT1* promoters are both are expressed in the endodermis and pericycle and to some extent in the cortex of Zn deficient roots or further inside the stele (only for *N*. *caerulescens*). While we never observed GUS expression in *A*. *thaliana* plants containing either a *pAtZIP4*::*GUS* construct or a *pNcZNT1*::*GUS* construct when exposed to sufficient Zn, we did see GFP expression in *pNcZNT1*::*eGFP A*. *thaliana* plants, though it appeared much weaker than in Zn deficient plants. In general, expression levels were higher for the *NcZNT1* promoter constructs compared to the *AtZIP4* constructs and for the *N*. *caerulescens* roots compared to the *A*. *thaliana* roots.

The Zn deficiency induced activity of the *AtZIP4* promoter in *A*. *thaliana* root endodermis and pericycle layers suggests that AtZIP4 acts to take up Zn in the endodermis, so it can pass the apoplastic root barrier formed by the Casparian Strips, and allows it to enter the stele symplast. Once in the endodermal/pericycle cells, it can be transported symplastically to the xylem companion cells, available for loading into the xylem for transport to the shoot. Since *AtZIP4* is not expressed in the epidermis, as was seen for the *AtIRT1* gene encoding a ZIP-transporter involved in Fe uptake from the soil [41], and it is also not expressed under ample Zn supply, it is unlikely to be important for the regular Zn uptake from the soil. Instead, it appears to provide an auxiliary Zn transport function only in case of a high (shoot) Zn demand. In contrast, the *NcZNT1* promoter is active under sufficient and deficient Zn supply in both species, and the activity is more prominent in the stele (pericycle and further inward) (Fig 5O–5W). This means both proteins have a similar function in their respective species, that is to ensure Zn is transported into the stele and kept there so it can be loaded into xylem and transported to aboveground parts. Probably due to the high constitutive demand of *N*. *caerulescens* shoots for Zn, the strict Zn deficient activity seen for the *AtZIP4* promoter is much less obvious for the *NcZNT1* promoter, possibly reflecting its general higher activity. Note that this Zn sufficient expression was never noticed when the *NcZNT1* promoter was driving GUS expression in *A*. *thaliana*, suggesting that the GUS assay is less sensitive than the GFP expression. The expression of *AtZIP4/NcZNT1* in the root endodermis and pericycle complies well with the expression of *AtHMA4/NcHMA4* in root xylem parenchyma. HMA4 is a plasma-membrane-located P-type ATPase Zn exporter, essential for root-to-shoot translocation of Zn [42]. During Zn deficiency, the combined action of ZIP4 and HMA4 will create a strong loading of apoplastic Zn across the endodermis into the stele and allow subsequent symplastic transport to the xylem parenchyma for loading into xylem for root-to-shoot transport [43]. *NcZNT1* and *AtZIP4* expression in the stele of older root tissues, where mineral uptake is minimal, would prevent Zn leakage and will ensure Zn availability for xylem loading and Zn supply to the shoots. This will be particularly important for the Zn hyperaccumulator *N*. *caerulescens*, which transports much more Zn to the shoots than *A*. *thaliana*. The roles and expression patterns of the *N*. *caerulescens NcHMA4* and *NcZNT1* genes are consistent with the observation that shoot metal hyperaccumulation is controlled by root processes [44].

The absence of *AtZIP4* promoter activity in *N*. *caerulescens* Zn sufficient roots, in which the *NcZNT1* promoter is active (Fig 4), has implications for understanding the evolution of Zn hyperaccumulation in *N*. *caerulescens*. It indicates that *N*. *caerulescens* still has a Zn deficiency sensing mechanism similar to that in *A*. *thaliana*, probably acting through bZIP19/bZIP23-like transcription factors [45]. However, the down-regulation of *AtZIP4* upon sufficient Zn supply in *A*. *thaliana* is much less for *NcZNT1* expression in *N*. *caerulescens*. This suggests that there is an additional level of regulation, probably independent of regulation through bZIP19/bZIP23, involving an additional transcription factor/transcription factor binding site combination. Such transcription factor function would be *N*. *caerulescens*-specific, as it does not act in *A*. *thaliana*. Something similar has been observed for the *At/NcHMA4* promoters, though the situation for these orthologous genes is more complicated as the *NcHMA4* gene is present in several tandem copies, compared to the single *AtHMA4* copy in *A*. *thaliana* [46, 47]. While endogenous activities of the *AtHMA4* and *NcHMA4* promoters (in their native species) are very similar, the heterologous activities of several *NcHMA4* promoters in *A*. *thaliana* confer ectopic expression of GUS [48]. So, for both genes, evolution of heavy metal adaptation has acted on transcription, although in many aspects, the transcriptional regulation is conserved. Altering transcription by simply expanding the copy number of a gene under selection to enhance transcription may be a good start, but in case tissue specificity or response to environmental cues is favoured over simply enhanced transcription, the more time-demanding route of promoter sequence and transcription factor binding evolution may prevail.

Another clue for the general conservation of transcriptional regulation was found in the analysis of 5’ deletions of the *AtZIP4* and *NcZNT1* promoters when fused to GUS (Fig 6). This showed that only deletion of regions containing two conserved palindromic *cis*-regulatory elements reduced GUS activity in *A*. *thaliana* (Fig 6). We previously reported that these *cis* elements, termed Zinc Deficiency Response Elements (ZDREs), are the binding sites for basic-region leucine zipper (bZIP) transcription factors bZIP19 and bZIP23 [22]. These transcription factors regulate a set of target genes, including *AtZIP4* as a first response to Zn deficiency. The same conserved ZDREs are found in orthologous promoters from related species *A*. *halleri*, *A*. *lyrata* and *C*. *pyrenaica* (S6 Fig). Apart from these proximal promoter regions, there is very poor sequence conservation when comparing these promoters. This is not very remarkable, as the deletion analysis showed that the active promoter does not stretch much further upstream than just beyond the most distal ZDRE (Fig 6).

A remarkable characteristic of Zn hyperaccumulators is the high expression of Zn homeostasis genes [11–13, 49, 50]. In non-hyperaccumulators these genes are mainly induced upon Zn deficiency and bZIP19 and bZIP23 are the known regulators of Zn deficiency responsive genes [22]. This may be taken to suggest that Zn hyperaccumulator roots are actually Zn deficient due to the high root-to-shoot Zn translocation. However, if that were the case, the *AtZIP4* promoter would be expected to be active in Zn sufficient *N*. *caerulescens* roots, which is not what we observed, neither when using GUS, nor GFP as marker gene (Figs 4 and 5). Instead it looks as if there is a higher basal activity of *NcZNT1* promoter compared to the *AtZIP4* promoter, which is more likely to be due to the action of an additional transcription factor, other than the bZIP19/bZIP23 proteins.

Ectopic expression of *NcZNT1* in the *p35S*::*NcZNT1* transgenics most likely disturbed Zn distribution over the plant and affected Zn use efficiency, as can be seen from the higher sensitivity of transgenics to Zn deficiency (S8A Fig). Nevertheless, transgenic plants accumulated more Zn and Cd than WT, which is consistent with a Zn and Cd transport ability of NcZNT1 and in line with results found with the ZNT1 orthologue from the related Ni hyperaccumulator species *Noccaea japonica* (formerly *Thlaspi japonica*) (NjZNT1), which transports Zn, Cd, and Mn into yeast [51]. However, heterologous expression in yeast might not properly reflect the protein function in plants. Although the transgenic *p35S*::*NcZNT1* lines also exhibited enhanced shoot Mn accumulation in Zn excess, both the enhanced Mn and Cd accumulation could be an indirect effect of altered Zn homeostasis in the transgenics.

Under excess Zn and Cd, Fe uptake is compromised in *A*. *thaliana* [13]. As some of the known Fe uptake transporters can transport Zn and Cd [52], they could indirectly play a role in Zn and Cd accumulation. Since we found a reduced Fe accumulation under Cd exposure in our transgenic lines compared to WT, we analysed the gene expression of known Fe transporters to find their possible role in indirect Zn and Cd accumulation in our transgenic lines. Fe-deficiency-responsive genes like *AtbHLH100*, *AtIRT1*, *AtIRT2* and *AtFRO2* were highly upregulated in both transgenic and WT lines under Zn and Cd excess, clearly showing that these lines experienced Fe deficiency, but the upregulation of *AtIRT1* and *AtFRO2*, the main genes involved in Fe uptake, is less in the transgenics upon Zn excess (Fig 11; S1 Table). Furthermore, higher expression of these known transporters could possibly mediate Zn and Cd accumulation. Particularly AtIRT1 was previously shown to transport Zn and Cd in addition to Fe in rhizodermal cells [52–54]. As we only examined transcription and not the actual presence of Fe transporters at the protein level, we cannot exclude that some of the additional Zn and Cd uptake, and probably all of the additional Mn uptake, in *p35S*::*NcZNT1* lines is due to the upregulation of the Fe deficiency responsive machinery and differences in protein levels in response to the ectopic expression of NcZNT1. The higher tolerance to excess Zn and Cd exposure of the transgenics compared to the WT plants is not obviously explained by differences in the transcription of the vacuolar Zn transporters *AtHMA3*, *AtMTP1* or *AtNRAMP3*, but the increased tolerance can be a combination of changing the distribution of Zn over different tissues, making it more equally distributed in comparison to the wild type, and the indirect effect this altered distribution may have on other Zn and Fe homeostasis genes. Only the small, but significant, differences in *AtMTP1* transcription, probably in response to ectopic expression and accumulation of Zn, are consistent with the observed differences in Zn excess tolerance.

*NcZNT1* has recently also been studied by Milner et al. [23]. They proposed *NcZNT1* to be involved in Zn uptake in root tissues and long distance transport. Our data are consistent with the role of *NcZNT1* in keeping higher influx into cells associated with xylem loading for root-to-shoot translocation, but not regarding its involvement in Zn uptake from the soil. Their study included the functional analysis of *NcZNT1* in yeast and in *A*. *thaliana* [23], which showed NcZNT1 did not transport Cd into yeast and that *p35S*::*NcZNT1* expressing *A*. *thaliana* lines were sensitive to excess Zn but not to Cd. However, we found there is a major difference in the *NcZNT1* cDNA sequence they used in their research, compared to the one we used. This difference leads back to earlier reports describing the initial identification of the *NcZNT1* gene [5, 17]. The *NcZNT1* cDNA used in [5, 23] was cloned from a plant originating from a natural population of *N*. *caerulescens* growing at the town of Prayon in Belgium (*NcZNT1-PR*). The *NcZNT1* cDNA used in this study was cloned from a plant originating from a natural population found close to the village of La Calamine (*NcZNT1-LC*), some 30 km distant from Prayon [17]. The *NcZNT1-PR* cDNA is only 14 bp shorter than the *NcZNT1-LC* cDNA, just missing the first ATG start codon found in the latter cDNA and thus predicted to result in an N-terminal truncated protein lacking 30 amino acids (Fig 1; S1 Fig; S2 Fig). The first ATG start codon found in *NcZNT1-LC* is also found in the *AtZIP4* cDNA sequence as well as in the (predicted) cDNA sequences of the *NcZNT1/AtZIP4* orthologues of *A*. *lyrata* (GenBank acc. no. XM_002892566), *Camelina sativa* (acc. nos. XM_010460175; XM_010493899; XM_010477683), *Capsella rubella* (acc. no. XM_006307604) and *Eutrema salsugineum* (acc. no. XM_006417336) (data not shown). Since it is so conserved, and the protein sequences of the before mentioned orthologues are as well, all predicted to have an N-terminal signalling sequence suggesting it to be targeted to the plasma membrane [17], we conclude that it is most likely that the published *NcZNT1-PR* cDNA sequence [5, 23] happens to be a partial cDNA, just lacking the original ATG start codon sequence. Lacking a proper N-terminus is likely to affect the normal function and cellular localization of the aberrant NcZNT1-PR protein. For instance, deletion of the 33 amino-acid N-terminus of the mammalian RGS4 protein, a GTPase-activating protein, resulted in loss of plasma membrane localization [55]. Mislocalization of the aberrant NcZNT1-PR protein upon ectopic expression might explain why we found transgenic *p35S*::*NcZNT1-LC*-expressing *A*. *thaliana* lines to be tolerant to excess Zn and Zn accumulation (Figs 7, 8 and 9), while Milner et al. found *p35S*::*NcZNT1-PR* lines to be Zn sensitive [23]. It may also explain why we did not observe GUS expression in *A*. *thaliana* expressing *NcZNT1-LC* promoter deletion fragments fused to GUS under Zn sufficient conditions, while such was found by Milner et al. [23]. As they considered the second ATG of *NcZNT1-PR* cDNA as the start codon, they inadvertently included the full first intron and part of the first exon as part of the promoter. Such could have affected the GUS expression. Though it is still conceivable that the PR genotype used to clone the *NcZNT1-PR* cDNA is making different transcripts, one with and one without the first ATG, we showed that the longer transcript, containing the first ATG, is actually made in PR and we confirmed the transcribed *NcZNT1* genomic regions of PR and LC to be nearly identical. Thus we conclude that the shorter, aberrant *NcZNT1-PR* cDNA used by [22], appears to be an artefact, probably the result of incomplete reverse transcription of a normal full-length *NcZNT1-PR* mRNA.

## Supporting Information

S1 Fig Comparison of predicted NcZNT1 and AtZIP4 proteins. The predicted protein sequences of *NcZNT1* from *N*. *caerulescens* accessions La Calamine (NcZNT1-LC) and Prayon (NcZNT1-PR) as deposited in GenBank (www.ncbi.nlm.nih.gov/nuccore/) (respectively AF275751.1, from LC, and AF133267.1, from PR), and *A*. *thaliana ZIP4* (AtZIP4; the At1g10970.1 gene model in www.arabidopsis.org) are compared. Identical amino acids are boxed in black. The alignment was performed using MultAlin (http://multalin.toulouse.inra.fr/multalin).
(TIF)

S2 Fig Comparison of full length *NcZNT1* genomic DNA sequences of *N*. *caerulescens* accessions La Calamine, Prayon, and Ganges. *NcZNT1* genomic DNA fragments were amplified from La Calamine (LC), Prayon (PR), and Ganges (GA) using primer pairs indicated with black lines. The blue arrow indicates part of the 5’ untranslated region (UTR). The red arrow indicates the predicted translational start codon (ATG), the blue bar the predicted translational stop codon (TAG). Four exons are indicated with black arrows and three introns are indicated with black dotted lines. GenBank numbers for the genomic DNA sequences are KU298431, KU298432 and KU298433 for resp. LC, PR and GA. The alignment was performed using MultAlin (http://multalin.toulouse.inra.fr/multalin).
(TIF)

S3 Fig Localization of GFP-tagged NcZNT1 in cowpea protoplasts. Construct *p35S*::*NcZNT1*:*GFP* was transiently expressed in cowpea protoplasts. Upon UV-illumination, expression of the NcZNT1-GFP fusion protein can be observed in the plasma membrane (green arrow). Due to the very high expression caused by the strong CaMV 35S promoter, there is additional GFP signal in the cytoplasm. There is no obvious GFP signal in organellar membranes, such as those of vacuoles or chloroplasts. Panel A shows the red auto-fluorescence of chloroplasts; panel B shows GFP florescence image; and panel C is the merged images of each set. Scale bars are indicated.
(TIF)

S4 Fig *NcZNT1* transcription in *N*. *caerulescens* and in *p35S*::*NcZNT1* expressing *A*. *thaliana*. (A) *N*. *caerulescens* plants were grown for four weeks in ½ Hoagland’s nutrient solution supplemented with 0.05 μM ZnSO_4_ (Zn deficiency), 2 and 10 μM ZnSO_4_ (sufficient Zn) and 1000 μM ZnSO_4_ (excess Zn). The *CLATHRIN* gene was used for cDNA normalization. Relative transcription levels (RTLs) were calculated, with the RTL of *NcZNT1* in shoots of Zn excess exposed plants set to 1. Error bars indicate the standard errors of the mean, n = 4. Different letters indicate significant differences (p<0.05) among plant types within treatments. (B) *NcZNT1* RTLs in *p35S*::*NcZNT1* expressing *A*. *thaliana* grown with sufficient Zn supply (2 μM ZnSO_4_). The *AtUBP6* gene was used for cDNA normalization. As expected, *NcZNT1* was not found to be transcribed in Col-0 wild-type plants. Error bars indicate the standard errors of the mean, n = 4. * indicates RTLs that are statistically significantly different from transcription in plants grown at 2 μM ZnSO_4_ (p< 0.05, Student’s t test).
(TIF)

S5 Fig Quantitative analysis of GUS expression in roots of transgenic *A*. *thaliana* plants expressing (A) *pAtZIP4*::*GUS* or (B) *pNcZNT1*::*GUS*. Two-week old seedlings were transferred to half Hoagland’s nutrient solution containing different Zn concentrations (no Zn added, 0.1, 0.2, 0.5, 1 and 2 μM ZnSO_4_). Roots were harvested every week, for 8 weeks, for quantitative GUS analysis.
(TIF)

S6 Fig *ZIP4*-orthologous promoters of *A*. *thaliana*, *N*. *caerulescens*, *C*. *pyrenaica*, *A*. *halleri* and *A*. *lyrata*. Sequences similar to *A*. *thaliana* are shown for the non-*A*. *thaliana* fragments in light (50–80% similarity) and dark grey (80–100% similarity) boxes, as are sequences of *A*. *thaliana* similar to *N*. *caerulescens*. Two conserved palindromic sequences found within 250 bp from the transcription start (+1) are indicated with dark grey boxes. The similar sequences found at the 5’ end of the promoter fragments represent sequences of the gene upstream of the *ZIP4* (orthologue).
(TIF)

S7 Fig Flowering times of transgenic *p35S*::*NcZNT1* and wild-type *A*. *thaliana* lines grown under Zn deficiency. Three independently transformed lines (NcZNT1-1, NcZNT1-2, NcZNT1-3) and Col wild-type (Col-WT) line were grown hydroponically on half Hoagland’s solution without Zn added to the medium for five weeks. (A) Flowering phenotype of *p35S*::*NcZNT1* plants. (B) Vegetative phenotype of comparable WT plants. (C) Flowering time in days after sowing of the three independently transformed *p35S*::*NcZNT1* lines compared to Col-WT (mean ± SE of 4 replicates). Different letters indicate significant differences (p<0.05) between genotypes (transgenic lines vs. wild-type) as determined by ANOVA.
(TIF)

S8 Fig Visible differences in plant phenotypes and Zn concentrations of transgenic *p35S*::*NcZNT1* and wild-type *A*. *thaliana* lines grown under Zn deficiency. Three independently transformed lines (NcZNT1-1, NcZNT1-2, NcZNT1-3) and Col wild-type (Col-WT) line were grown hydroponically in half Hoagland’s media supplemented with 0.05 μM ZnSO_4_ (Zn deficiency) for four weeks. (A) Visible phenotypes of *p35S*::*NcZNT1* and Col-WT plants. (B) Zn concentration in shoot and root (μmoles/g DW) (mean ± SE of 4 replicates). Different letters indicate significant differences (p<0.05) among plant types within treatments (transgenic lines and wild-type).
(TIF)

S9 Fig Fe and Mn concentrations of transgenic *p35S*::*NcZNT1* and wild-type *A*. *thaliana* lines grown at excess Zn and Cd supply. Three independently transformed lines (NcZNT1-1, NcZNT1-2, NcZNT1-3) and Col wild-type (Col WT) were grown hydroponically for four weeks on half Hoagland’s media, including sufficient Zn (2 μM ZnSO_4_), excess Zn (60 μM ZnSO_4_) and Cd (2 μM CdSO_4_). (A) Fe concentration in shoots (μmoles/g DW) (B) and in roots (μmoles/g DW) (C) Mn concentration in shoots (μmoles/g DW) (D) and in roots (μmoles/g DW) (mean ± SE of 4 replicates). Different letters indicate significant differences (p<0.05) among plant types (transgenic lines and wild-type) and treatments.
(TIF)

S1 Movie Movie comprised of stacked confocal images corresponding to the cowpea protoplast transiently expressing *p35S*::*NcZNT1*:*GFP*, shown in S3 Fig. (AVI)

S1 Table Relative transcription levels of metal homeostasis genes (*AtBHLH100*, *AtIRT1*, *AtIRT2*, *AtFRO2*, *AtHMA4*, *AtHMA3*, *AtFRD3*, *AtMTP1*, *AtNRAMP3* and *AtYSL3*) in shoots and roots of *p35S*::*NcZNT1* transgenic (ZNT1) and Col wild-type (WT) *A*. *thaliana* plants, in response to sufficient Zn (2 μM ZnSO_4_; SuffZn), excess Zn (60 μM ZnSO_4_; ExcessZn) and Cd (2 μM CdSO_4_) after two weeks of metal exposure, corresponding to Fig 6. Transcription is relative to that in WT shoots of plants grown with sufficient Zn, which was set to relative transcription level (RTL) = 1. *AtUBP6* (At1g51710) was used as reference gene to normalize cDNA samples. Different letters indicate significant differences in RTL of the respective gene comparing ZNT1 and WT lines grown in indicated treatments (p<0.05, ANOVA, Least Significant Difference Test) (mean ± SE of 4 replica).
(PDF)
