# Supplementary figures and images for: Expression of the ZNT1 Zinc Transporter from the Metal Hyperaccumulator Noccaea caerulescens Confers Enhanced Zinc and Cadmium Tolerance and Accumulation to Arabidopsis thaliana
Source: PLoS One. 2016 Mar 1;11(3):e0149750. doi: 10.1371/journal.pone.0149750 (PMC4773103; doi:10.1371/journal.pone.0149750)

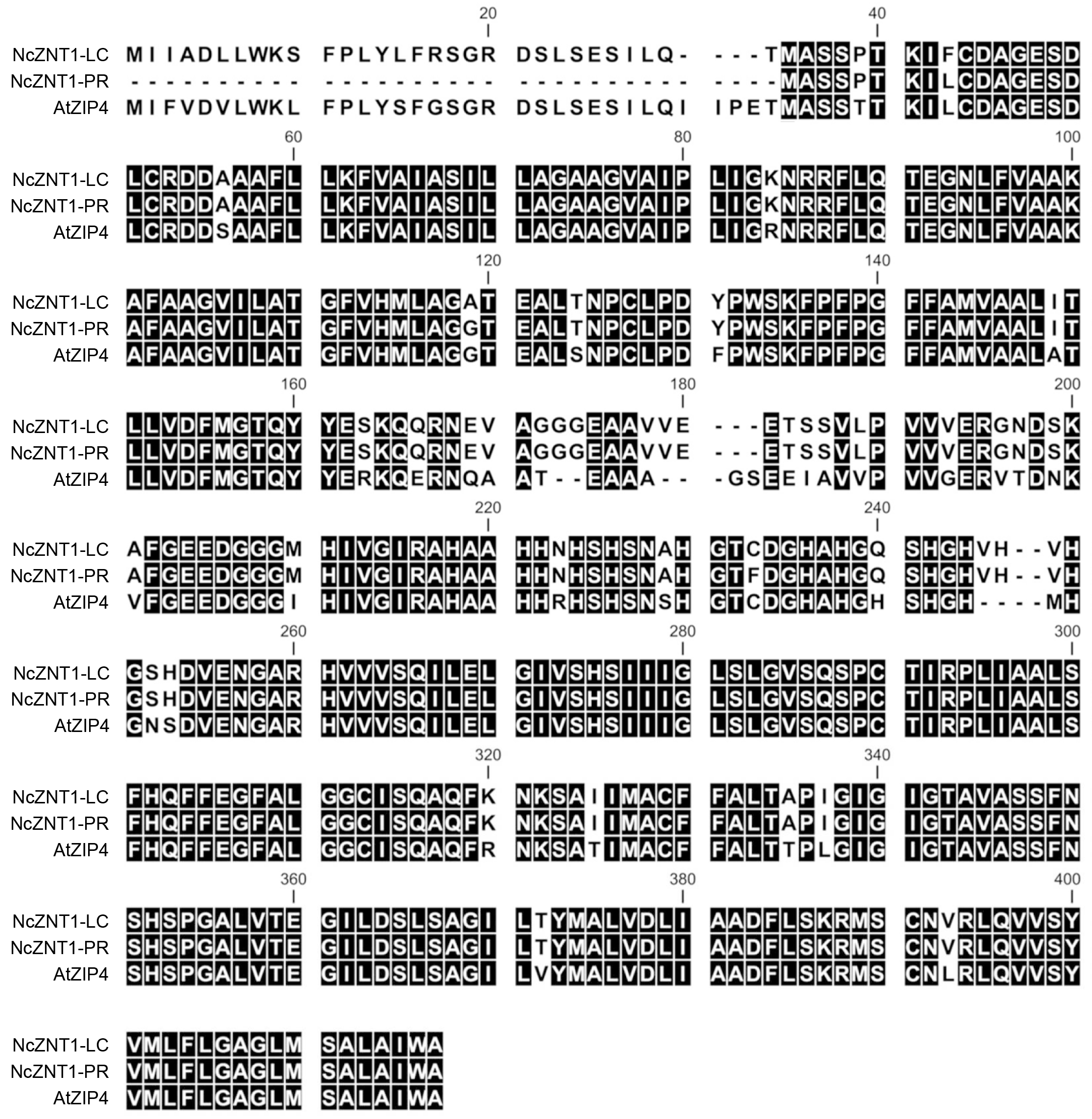

Supplement: S1 Fig — The predicted protein sequences of NcZNT1 from N. caerulescens accessions La Calamine (NcZNT1-LC) and Prayon (NcZNT1-PR) as deposited in GenBank (www.ncbi.nlm.nih.gov/nuccore/) (respectively AF275751.1, from LC, and AF133267.1, from PR), and A. thaliana ZIP4 (AtZIP4; the At1g10970.1 gene model in www.arabidopsis.org) are compared. Identical amino acids are boxed in black. The alignment was performed using MultAlin (http://multalin.toulouse.inra.fr/multalin). (TIF) [file pone.0149750.s001.tif]

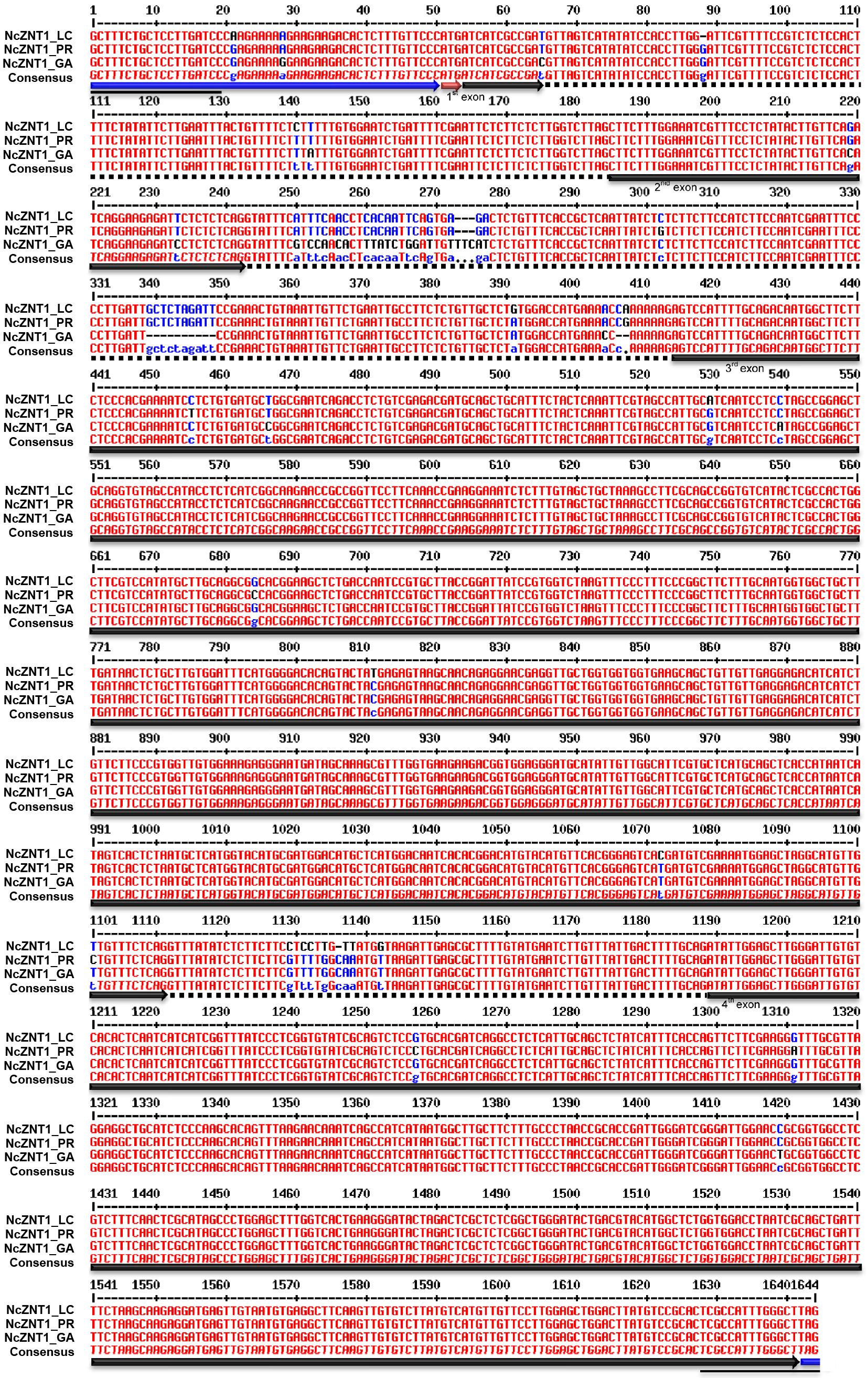

Supplement: S2 Fig — NcZNT1 genomic DNA fragments were amplified from La Calamine (LC), Prayon (PR), and Ganges (GA) using primer pairs indicated with black lines. The blue arrow indicates part of the 5’ untranslated region (UTR). The red arrow indicates the predicted translational start codon (ATG), the blue bar the predicted translational stop codon (TAG). Four exons are indicated with black arrows and three introns are indicated with black dotted lines. GenBank numbers for the genomic DNA sequences are KU298431, KU298432 and KU298433 for resp. LC, PR and GA. The alignment was performed using MultAlin (http://multalin.toulouse.inra.fr/multalin). (TIF) [file pone.0149750.s002.tif]

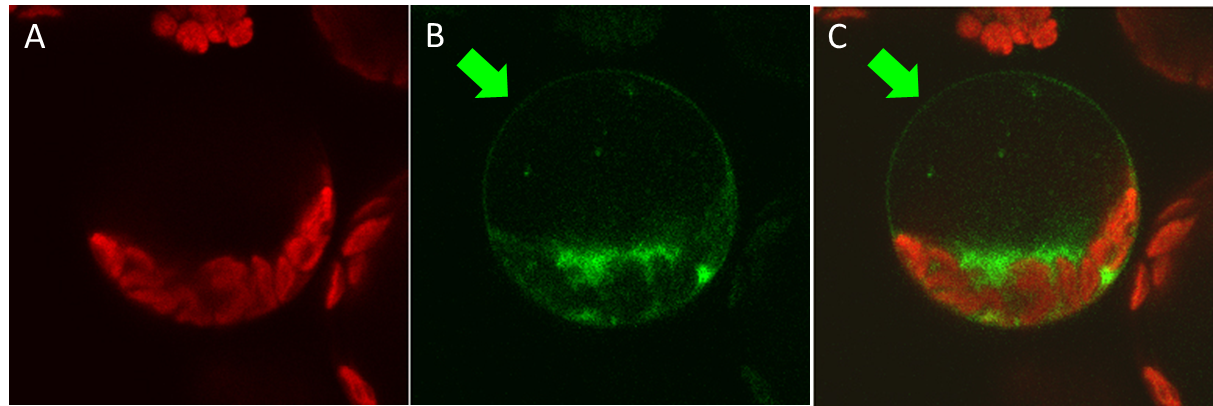

Supplement: S3 Fig — Construct p35S::NcZNT1:GFP was transiently expressed in cowpea protoplasts. Upon UV-illumination, expression of the NcZNT1-GFP fusion protein can be observed in the plasma membrane (green arrow). Due to the very high expression caused by the strong CaMV 35S promoter, there is additional GFP signal in the cytoplasm. There is no obvious GFP signal in organellar membranes, such as those of vacuoles or chloroplasts. Panel A shows the red auto-fluorescence of chloroplasts; panel B shows GFP florescence image; and panel C is the merged images of each set. Scale bars are indicated. (TIF) [file pone.0149750.s003.tif]

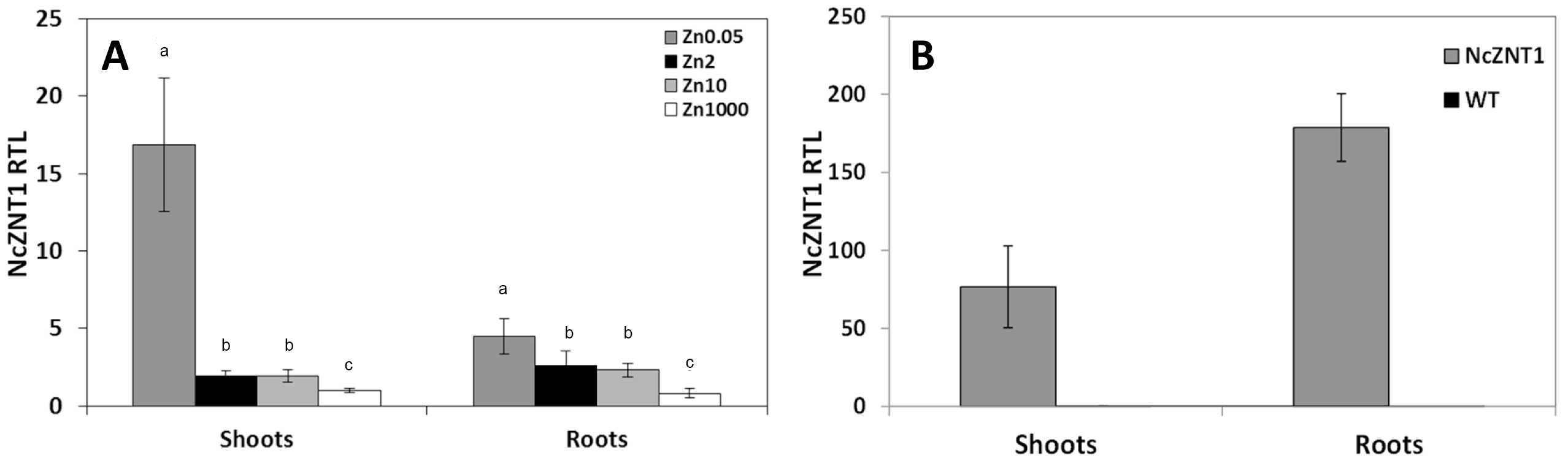

Supplement: S4 Fig — (A) N. caerulescens plants were grown for four weeks in ½ Hoagland’s nutrient solution supplemented with 0.05 μM ZnSO4 (Zn deficiency), 2 and 10 μM ZnSO4 (sufficient Zn) and 1000 μM ZnSO4 (excess Zn). The CLATHRIN gene was used for cDNA normalization. Relative transcription levels (RTLs) were calculated, with the RTL of NcZNT1 in shoots of Zn excess exposed plants set to 1. Error bars indicate the standard errors of the mean, n = 4. Different letters indicate significant differences (p<0.05) among plant types within treatments. (B) NcZNT1 RTLs in p35S::NcZNT1 expressing A. thaliana grown with sufficient Zn supply (2 μM ZnSO4). The AtUBP6 gene was used for cDNA normalization. As expected, NcZNT1 was not found to be transcribed in Col-0 wild-type plants. Error bars indicate the standard errors of the mean, n = 4. * indicates RTLs that are statistically significantly different from transcription in plants grown at 2 μM ZnSO4 (p< 0.05, Student’s t test). (TIF) [file pone.0149750.s004.tif]

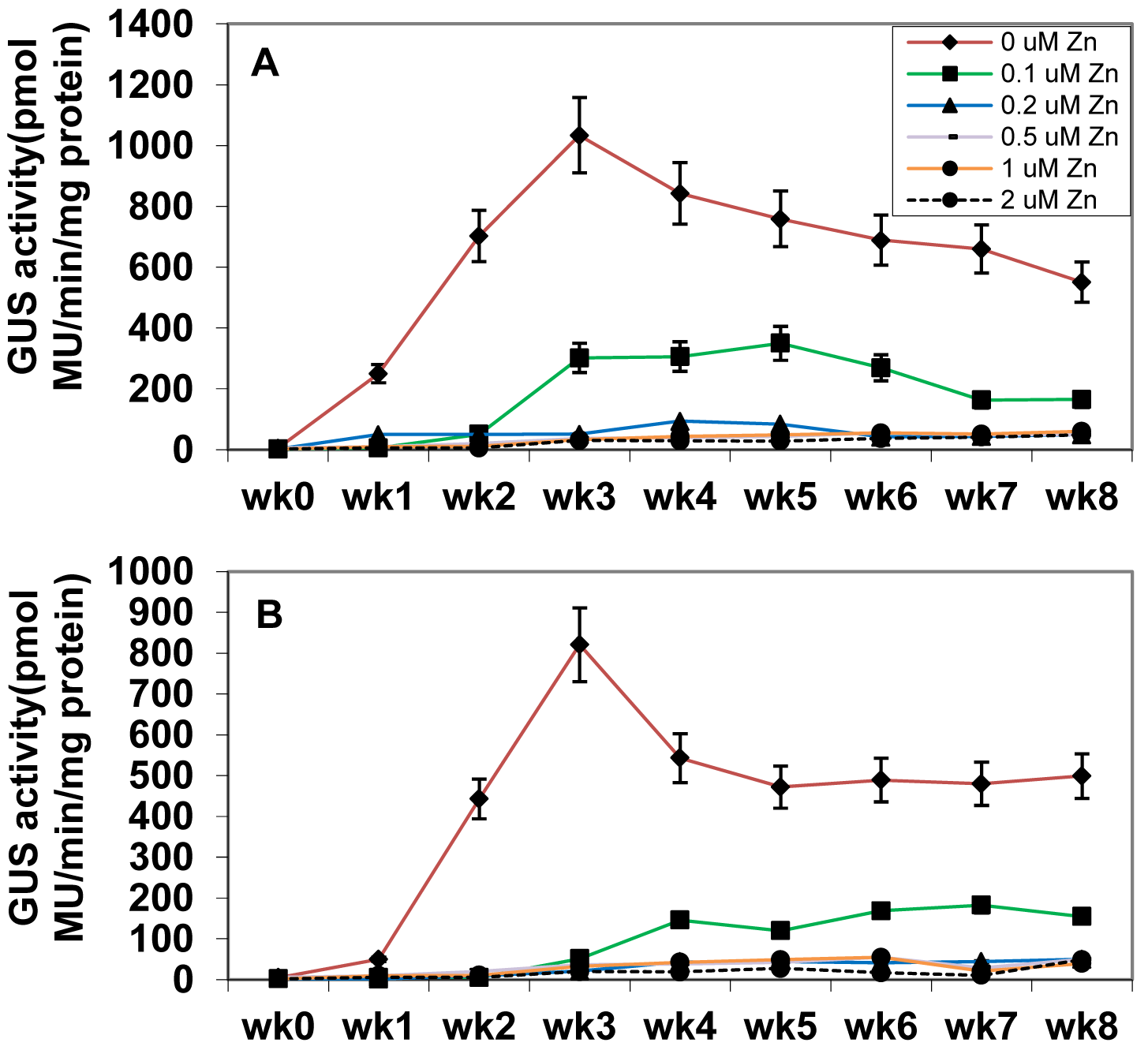

Supplement: S5 Fig — Quantitative analysis of GUS expression in roots of transgenic A. thaliana plants expressing (A) pAtZIP4::GUS or (B) pNcZNT1::GUS. Two-week old seedlings were transferred to half Hoagland’s nutrient solution containing different Zn concentrations (no Zn added, 0.1, 0.2, 0.5, 1 and 2 μM ZnSO4). Roots were harvested every week, for 8 weeks, for quantitative GUS analysis. (TIF) [file pone.0149750.s005.tif]

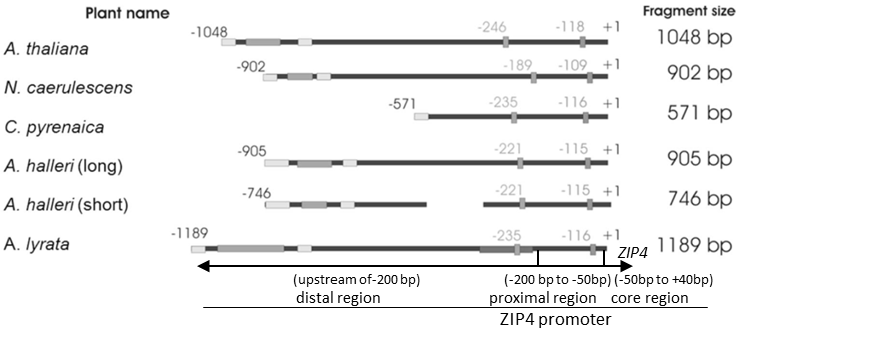

Supplement: S6 Fig — Sequences similar to A. thaliana are shown for the non-A. thaliana fragments in light (50–80% similarity) and dark grey (80–100% similarity) boxes, as are sequences of A. thaliana similar to N. caerulescens. Two conserved palindromic sequences found within 250 bp from the transcription start (+1) are indicated with dark grey boxes. The similar sequences found at the 5’ end of the promoter fragments represent sequences of the gene upstream of the ZIP4 (orthologue). (TIF) [file pone.0149750.s006.tif]

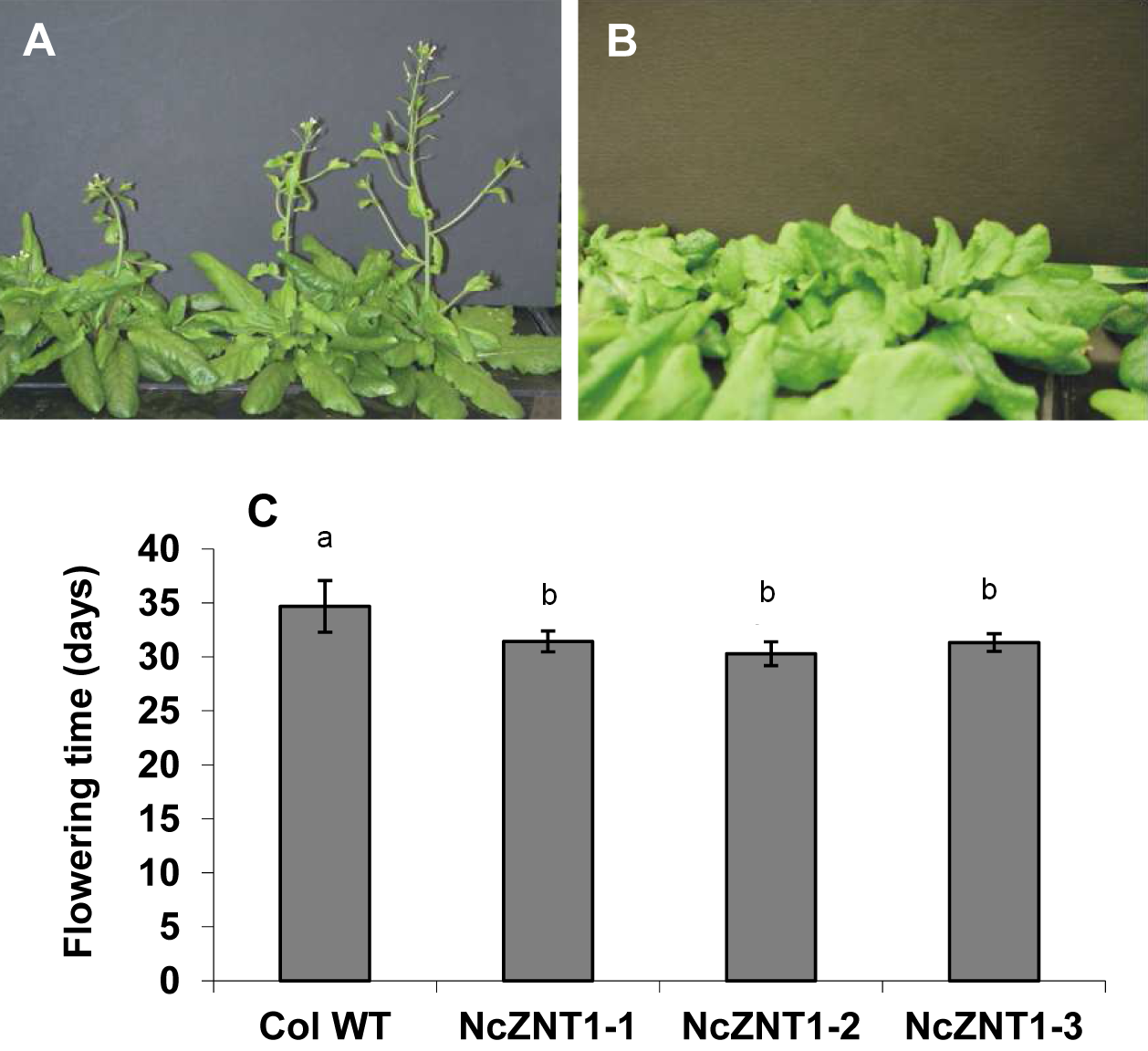

Supplement: S7 Fig — Three independently transformed lines (NcZNT1-1, NcZNT1-2, NcZNT1-3) and Col wild-type (Col-WT) line were grown hydroponically on half Hoagland’s solution without Zn added to the medium for five weeks. (A) Flowering phenotype of p35S::NcZNT1 plants. (B) Vegetative phenotype of comparable WT plants. (C) Flowering time in days after sowing of the three independently transformed p35S::NcZNT1 lines compared to Col-WT (mean ± SE of 4 replicates). Different letters indicate significant differences (p<0.05) between genotypes (transgenic lines vs. wild-type) as determined by ANOVA. (TIF) [file pone.0149750.s007.tif]

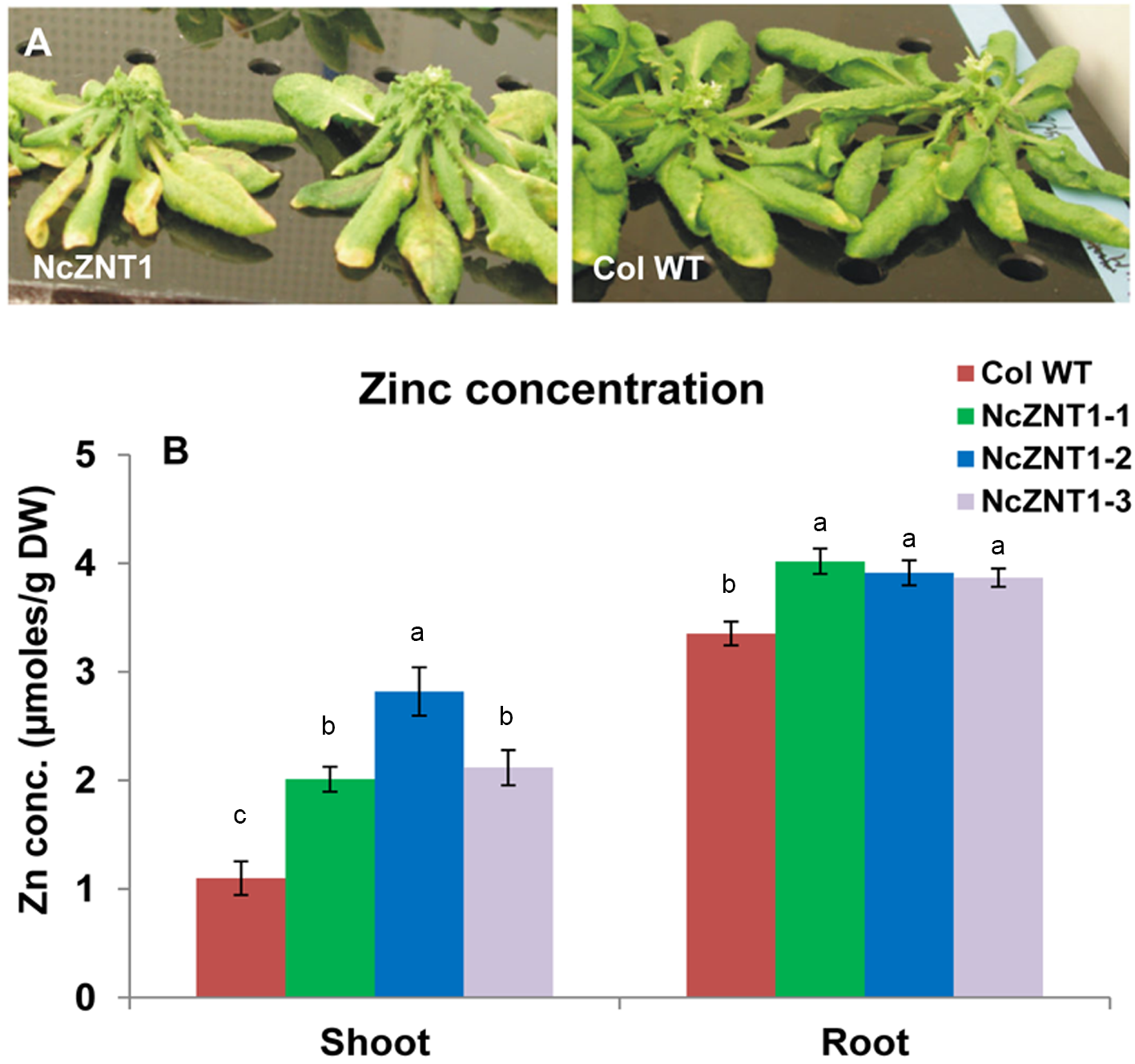

Supplement: S8 Fig — Three independently transformed lines (NcZNT1-1, NcZNT1-2, NcZNT1-3) and Col wild-type (Col-WT) line were grown hydroponically in half Hoagland’s media supplemented with 0.05 μM ZnSO4 (Zn deficiency) for four weeks. (A) Visible phenotypes of p35S::NcZNT1 and Col-WT plants. (B) Zn concentration in shoot and root (μmoles/g DW) (mean ± SE of 4 replicates). Different letters indicate significant differences (p<0.05) among plant types within treatments (transgenic lines and wild-type). (TIF) [file pone.0149750.s008.tif]

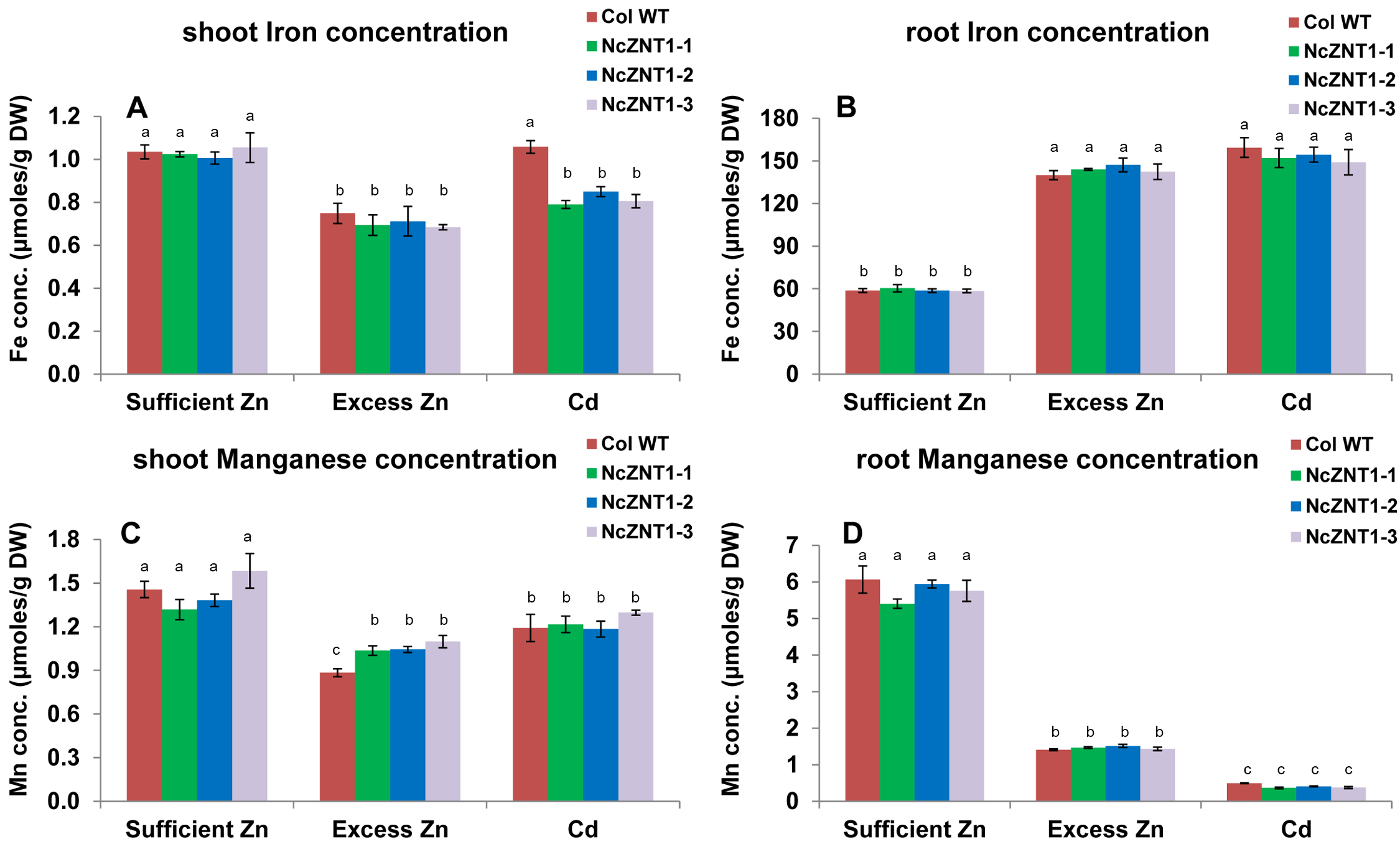

Supplement: S9 Fig — Three independently transformed lines (NcZNT1-1, NcZNT1-2, NcZNT1-3) and Col wild-type (Col WT) were grown hydroponically for four weeks on half Hoagland’s media, including sufficient Zn (2 μM ZnSO4), excess Zn (60 μM ZnSO4) and Cd (2 μM CdSO4). (A) Fe concentration in shoots (μmoles/g DW) (B) and in roots (μmoles/g DW) (C) Mn concentration in shoots (μmoles/g DW) (D) and in roots (μmoles/g DW) (mean ± SE of 4 replicates). Different letters indicate significant differences (p<0.05) among plant types (transgenic lines and wild-type) and treatments. (TIF) [file pone.0149750.s009.tif]
