# Supplementary material for: Expression of the ZNT1 Zinc Transporter from the Metal Hyperaccumulator Noccaea caerulescens Confers Enhanced Zinc and Cadmium Tolerance and Accumulation to Arabidopsis thaliana
Source: PLoS One. 2016 Mar 1;11(3):e0149750. doi: 10.1371/journal.pone.0149750 (PMC4773103; doi:10.1371/journal.pone.0149750)
Supplement: S1 Table — Transcription is relative to that in WT shoots of plants grown with sufficient Zn, which was set to relative transcription level (RTL) = 1. AtUBP6 (At1g51710) was used as reference gene to normalize cDNA samples. Different letters indicate significant differences in RTL of the respective gene comparing ZNT1 and WT lines grown in indicated treatments (p<0.05, ANOVA, Least Significant Difference Test) (mean ± SE of 4 replica). (PDF) [file pone.0149750.s011.pdf]

	WT SuffZn Shoot	ZNT1 SuffZn Shoot	WT ExcessZn Shoot	ZNT1 ExcessZn Shoot	WT Cd Shoot	ZNT1 Cd Shoot	WT SuffZn Root	ZNT1 SuffZn Root	WT ExcessZn Root	ZNT1 ExcessZn Root	WT Cd Root	ZNT1 Cd Root
AtBHLH100	a 1.0 ±0.1	a 0.8 ±0.3	f 2760 ±362	c 698 ±78	h 13046 ±595	i 16240 ±1919	b 5.5 ±0.7	b 4.4 ±1.7	g 5377 ±760	g 4274 ±625	d 1127 ±66	e 1685 ±222
AtIRT1	ab 1.0 ±0.2	a 0.5 ±0.3	b 3.0 ±0.5	a 0.4 ±0.0	c 5.9 ±1.5	c 6.2 ±0.7	e 1286 ±91	d 175 ±44	h 81904 ±15916	g 11084 ±1771	g 11651 ±2095	f 2389 ±86
AtIRT2	a 1.0 ±0.2	a 1.0 ±0.3	a 1.3 ±0.1	c 4.2 ±0.1	a 1.3 ±0.0	b 2.3 ±0.1	d 18 ±2.2	d 23 ±6.4	g 3661 ±494	f 1082 ±131	e 597 ±121	ef 850 ±52
AtFRO2	a 1.0 ±0.1	a 0.8 ±0.3	a 1.5 ±0.2	a 1.0 ±0.3	b 5.7 ±0.1	c 8.2 ±0.9	e 123 ±8.2	d 47 ±7.5	g 6018 ±665	f 2650 ±605	e 129 ±5.7	e 141 ±4.9
AtHMA4	a 1.0 ±0.1	a 0.8 ±0.0	a 0.7 ±0.1	a 0.7 ±0.2	a 0.6 ±0.1	a 0.7 ±0.0	c 9.5 ±0.9	d 14 ±1.1	c 10 ±0.6	b 6.4 ±0.5	b 7.8 ±1.0	b 6.9 ±0.5
AtHMA3	a 1.0 ±0.1	a 1.1 ±0.6	b 2.0 ±0.1	ab 1.6 ±0.6	a 0.7 ±0.1	a 1.1 ±0.3	c 207 ±2.0	e 412 ±32	f 792 ±114	d 295 ±8.7	d 284 ±37	d 271± 2.3
AtFRD3	a 1.0 ±0.2	a 2.0 ±1.1	a 1.6 ±0.1	a 1.5 ±0.1	b 6.2 ±0.6	b 5.0 ±0.3	g 671 ±60	e 379 ±66	f 598 ±4.1	d 159 ±28	cd 114 ±23	c 86 ±7.6
AtMTP1	c 1.0 ±0.1	ab 0.5 ±0.1	a 0.4 ±0.0	bc 0.7 ±0.1	ab 0.5 ±0.0	c 1.1 ±0.1	e 2.2 ±0.3	e 2.6 ±0.4	c 1.2 ±0.1	d 1.7 ±0.1	bc 0.8 ±0.1	c 1.3 ±0.2
AtNRAMP3	a 1.0 ±0.1	a 0.9 ±0.0	a 1.0 ±0.1	a 0.9 ±0.2	a 0.9 ±0.1	a 0.8 ±0.1	b 2.0 ±0.1	b 2.1 ±0.2	b 2.0 ±0.3	c 2.9 ±0.3	a 1.1 ±0.1	a 1.0 ±0.0
AtYSL3	cd 1.0 ±0.1	cd 1.1 ±0.0	a 0.3 ±0.0	ab 0.4 ±0.1	ab 0.4 ±0.0	ab 0.5 ±0.1	cd 1.1 ±0.1	d 1.2 ±0.1	a 0.3 ±0.0	b 0.6 ±0.0	b 0.6 ±0.1	bc 0.7 ±0.0
